# c-di-GMP modulates type IV MSHA pilus retraction and surface attachment in *Vibrio cholerae*

**DOI:** 10.1038/s41467-020-15331-8

**Published:** 2020-03-25

**Authors:** Kyle A. Floyd, Calvin K. Lee, Wujing Xian, Mahmoud Nametalla, Aneesa Valentine, Benjamin Crair, Shiwei Zhu, Hannah Q. Hughes, Jennifer L. Chlebek, Daniel C. Wu, Jin Hwan Park, Ali M. Farhat, Charles J. Lomba, Courtney K. Ellison, Yves V. Brun, Javier Campos-Gomez, Ankur B. Dalia, Jun Liu, Nicolas Biais, Gerard C. L. Wong, Fitnat H. Yildiz

**Affiliations:** 1 Department of Microbiology and Environmental Toxicology, University of California – Santa Cruz, 1156 High St., BioMed 245, Santa Cruz, CA 95064 USA; 2 Departments of Bioengineering, Chemistry and Biochemistry, California Nano Systems Institute, University of California – Los Angeles, 420 Westwood Plaza, Room 5121 Engineering V, Los Angeles, CA 90095 USA; 3 Department of Biology, Brooklyn College, Room 307NE, 2900 Bedford Ave., Brooklyn, NY 11210 USA; 4 CUNY Graduate Center, 365 5th Ave., New York, NY 10016 USA; 5 Department of Microbial Pathogenesis, Yale University, 840 West Campus Drive, Advanced Biosciences Center 211, West Haven, CT 06516 USA; 6 Department of Biology, Indiana University – Bloomington, 1001 East Third St., Jordan Hall 469A, Bloomington, IN 47405 USA; 7 Department of Microbiology, Infectious Diseases, and Immunology, Faculty of Medicine, University of Montreal, Pavillon Roger-Gaudry, 2900, boulevard Édouard-Montpetit, C.P. 6128, Succursale Centre−ville, Montréal, QC H3C 3J7 Canada; 8 Cystic Fibrosis Research Center, University of Alabama at Birmingham, 1918 University Blvd., MCLM 702, Birmingham, AL 35233 USA; 9 Present Address: Lewis-Sigler Institute for Integrative Genomics, Princeton University, 355 Thomas Laboratory, Washington Road, Princeton, NJ 08544 USA

**Keywords:** Cell adhesion, Cellular microbiology, Biofilms, Pathogens

## Abstract

Biofilm formation by *Vibrio cholerae* facilitates environmental persistence, and hyperinfectivity within the host. Biofilm formation is regulated by 3’,5’-cyclic diguanylate (c-di-GMP) and requires production of the type IV mannose-sensitive hemagglutinin (MSHA) pilus. Here, we show that the MSHA pilus is a dynamic extendable and retractable system, and its activity is directly controlled by c-di-GMP. The interaction between c-di-GMP and the ATPase MshE promotes pilus extension, whereas low levels of c-di-GMP correlate with enhanced retraction. Loss of retraction facilitated by the ATPase PilT increases near-surface roaming motility, and impairs initial surface attachment. However, prolonged retraction upon surface attachment results in reduced MSHA-mediated surface anchoring and increased levels of detachment. Our results indicate that c-di-GMP directly controls MshE activity, thus regulating MSHA pilus extension and retraction dynamics, and modulating *V. cholerae* surface attachment and colonization.

## Abstract (web summary)

Biofilm formation by *Vibrio cholerae* is regulated by c-di-GMP and requires the type IV MSHA pilus. Here, Floyd et al. show that the MSHA pilus is a dynamic system, and that both extension and retraction are directly controlled by c-di-GMP via regulation of activity of the extension ATPase MshE.

## Introduction

*Vibrio cholerae*, is a facultative human pathogen and etiologic agent of the gastrointestinal diarrheal disease cholera^1,2^. *V. cholerae* is a natural inhabitant of aquatic environments, and as such organizes into multicellular biofilm communities that enhance environmental survival^3–8^. Moreover, ingestion of biofilm particles greater than 20 μm in size results in exacerbated disease pathology, demonstrating that *V. cholerae* biofilms represent a significant public health threat^9–13^.

Biofilm formation starts with the attachment of a bacterium to a surface. Bacteria can utilize a variety of cell-surface structures to facilitate initial surface interactions. Attachment of *V. cholerae* O1 and O139 isolates, to abiotic surfaces is requisite upon production of the type IV mannose-sensitive hemagglutinin (MSHA) pilus^4,14–19^. Type IV pili are dynamic macromolecular structures ubiquitous among both Gram-negative and Gram-positive bacteria, as well as archaea^20,21^. In Gram-negative bacteria, the type IV pilus is comprised of a cell envelope spanning multi-protein complex that allows for elaboration of the pilus on the cell surface^21^. Elaboration of the pilus structure, comprised primarily of a single major pilin protein, is facilitated by a polymerization/extension ATPase^21^. Many systems also contain a secondary depolymerization/retraction ATPase, which functions to retract the pilus from the cell surface^21^. Cycles of pilus extension and retraction have been shown to facilitate surface attachment, as well as surface-associated twitching motility within many bacterial species^22–26^. Despite the vital role and importance of the MSHA pilus in *V. cholerae* surface attachment, the dynamics of MSHA extension and retraction and their consequence on biofilm formation remain to be elucidated.

Bacterial surface attachment is a highly regulated process, though the role of intracellular signaling cascades in mediating pilus activity are poorly understood. In many bacterial species, surface attachment and biofilm formation are regulated by the small molecule secondary messenger 3’,5’-cyclic diguanylate monophosphate (c-di-GMP). Generation and degradation of c-di-GMP is facilitated by enzymes called diguanylate cyclases (DGCs) and phosphodiesterases (PDEs), respectively^27–29^. Upon generation, c-di-GMP interacts with downstream receptors to govern cellular processes, including motility and biofilm formation^13,27–29^. We have previously characterized the MSHA polymerization ATPase, MshE, as a high-affinity c-di-GMP receptor^18,30,31^. MshE harbors a novel c-di-GMP-binding motif that is also present in other ATPases associated with type II secretion and type IV pilus systems^18,30,31^. Our initial studies suggested that c-di-GMP interacts with MshE to promote MSHA production, and facilitate the transition of *V. cholerae* from motile to surface-attached cells through modulation of near-surface motility and attachment^18^.

Here, we present a model for the direct regulation of *V. cholerae* surface attachment through c-di-GMP-mediated modulation of MSHA pilus extension/retraction dynamics. We demonstrate that MSHA are dynamic retractile type IV pili, and that retraction is facilitated via the PilT depolymerization ATPase. Direct visualization of MSHA production confirms that c-di-GMP-binding to MshE promotes extension activity, and elaboration of pili on the cell surface. We additionally observe that the dynamic activity of MSHA extension and retraction is optimized directly by c-di-GMP-mediated regulation of MshE activity. Reduced intracellular levels of c-di-GMP were found to correlate with enhanced levels of MSHA retraction, consistent with decreased c-di-GMP-dependent MshE activity. Similarly, alteration of MshE to a “constitutively active” state not only enhanced MSHA extension, but also enhanced rates of retraction, suggesting that c-di-GMP regulates the optimal conformational state of MshE to mitigate proper extension/retraction dynamics. High-speed cell tracking illustrates that attenuation of MSHA extension/retraction activity impairs near-surface motility and surface attachment, while prolonged periods of retraction after surface attachment results in increased levels of detachment. Finally, we observe that alteration of MSHA activity dictates the ability of *V. cholerae* to persist within a model of biofilm competition. Taken together, these results indicate that c-di-GMP-mediated MSHA pilus extension/retraction activity is facilitated via the regulation of MshE function, and suggest that this multifaceted orchestration of pilus dynamics is vital for *V. cholerae* surface attachment and biofilm formation.

## Results

### MSHA pili are distributed laterally along the cell body

**Fig. 1:**
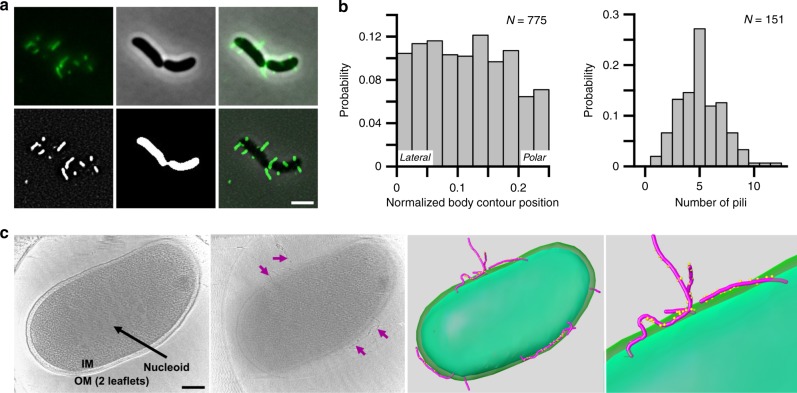
MSHA pili are laterally localized along the cell body. **a** Representative images of surface-associated *mshA*^*T70C*^ cells labeled with AlexaFluor 488 C_5_ maleimide dye for visualization of MSHA pili. Raw images obtained are shown in: upper left panel—fluorescence channel, upper middle panel—phase-contrast channel, and upper right panel—overlay. Processed images shown in: lower left panel—filtered-fluorescence channel, lower middle panel—binary image of phase-contrast channel, lower right panel—overlay of filtered-fluorescence and phase-contrast channels. Images representative of three independent analyses. Scale bar = 2 μm. **b** Probability of pilus distribution (*n* = 775 single cells) on the cell surface observed, and distribution of the average number of pili per cell (*n* = 151 single cells) observed from agarose-pad associated cells. **c** Imaging the whole-cell suing Cryo-ET and 3D segmentation. Far Left Panel—The central slice of tomograms of *mshA*^*T70C*^ whole-cell. Outer membrane two leaflets are visible. Nucleoid area is labeled in a white array. Middle Left Panel—Another slice from the same tomogram as shown in A, shows MSHA pili (marked with purple arrows) coated with the anti-MshA antibody. Middle Right Panel—3D segmentation of far left and middle left panels, with MSHA pili (purple) coated with anti-MshA antibody (yellow dots). Far Right Panel—Zoom-in image of 3D segmentation to show antibody-coating on the surface of pili. Colors same as in middle right panel. 3D segmentation performed from a single Cro-ET analysis, with Cryo-ET analysis performed on multiple individual *V. cholerae* cells. Scale bar = 200 nm.

To define the molecular basis of surface attachment using MSHA pili in *V. cholerae* O1 El Tor strain A1552, we employed direct visualization via thiol-reactive fluorescent dyes, through chromosomal insertion of *mshA* with a point mutation at amino acid residue 70 (*mshA*^*T70C*^, Fig. 1a and Supplementary Fig. 1a)^22^. Production of MSHA in the *mshA*^*T70C*^ strain was comparable to WT as determined by MSHA-specific hemagglutination (HA) assay (Supplementary Fig. 1b). Representative output of the MSHA-specific HA assay is shown in Supplementary Fig. 2. Visualization of the *mshA*^*T70C*^ strain demonstrates MSHA pili to be laterally distributed along the cell body (Fig. 1a, b, and Supplementary Fig. 1a, c). Lateral distribution of MSHA was validated through analysis using cryo-electron tomography (Fig. 1c, Supplementary Fig. 3, and Supplementary Movie 1). Quantification of the number of pili per cell shows a distribution between 1–12 pili per cell, with an average of 5.03 ± 2.02 pili under the conditions examined (Fig. 1b).

### MSHA pili are dynamic retractile macromolecular complexes

**Fig. 2:**
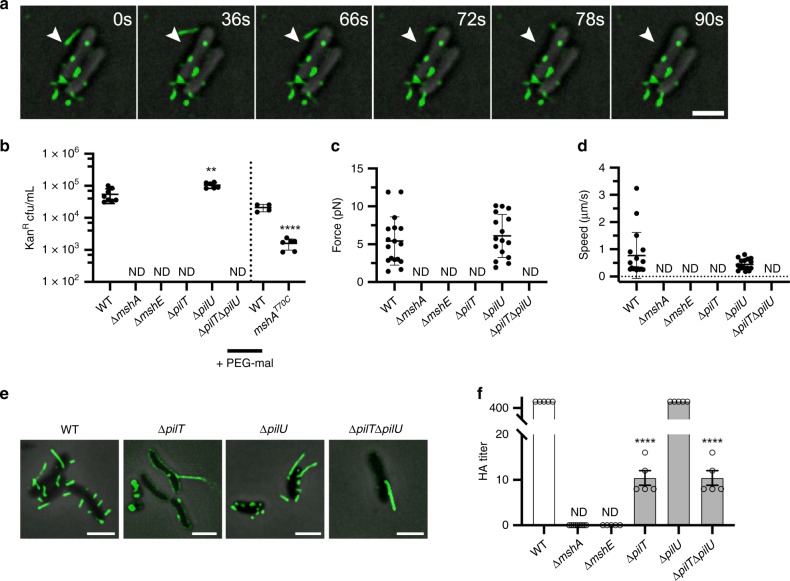
MSHA are dynamic retractile pili, and retraction is requisite upon the PilT ATPase. **a** Time-lapse visualization of MSHA retraction in surface-associated *mshA*^*T70C*^ cells labeled with AlexaFluor 594 C_5_ maleimide. Images were collected every 6 seconds over 10 min. White arrow in all images marks the tip of the retracting pilus at t = 0. Numerous retraction events were observed over multiple biological replicates (see Supplementary Movies). Scale bar = 2 μm. **b** Transduction of VGJΦ is dependent upon production of MSHA pili. Individual data points plotted with line at the mean and error bars representing the standard deviation. Biological replicates: WT *n* = 8, Δ*mshA* *n* = 6, Δ*mshE* *n* = 6, Δ*pilT* *n* = 6, Δ*pilU* *n* = 6, Δ*pilT*Δ*pilU* *n* = 6. Statistical analysis: unpaired two-tailed Student’s *t*-Test, **p = 0.0024. ND no detected kanamycin-resistant colonies. Data to the right of Dashed-line: WT and *mshA*^*T70C*^ cells treated with 4.2 mM MeOH-PEG-mal in DMSO prior to introduction of VGJΦ. Individual data points plotted with line at the mean and error bars representing the standard deviation. Biological replicates: WT *n* = 4, *mshA*^*T70C*^ *n* = 6. Statistical analysis, unpaired two-tailed Students *t*-Test, ****p ≤ 0.0001. Source data provided as a Source Data file. **c** Average force of MSHA retraction determined by micropillars assay. Individual data points plotted with line at mean and error bars representing the standard deviation. ND no retraction events detected. Biological replicates: WT *n* = 17 and Δ*pilU* *n* = 16. Statistical analysis of WT vs. Δ*pilU*, unpaired two-tailed Students *t*-Test, not significant *p* = 0.5291. Source data provided as a Source Data file. **d** Average speed of MSHA retraction determined by micropillar assay. Individual data points plotted with line at mean and error bars representing the standard deviation. ND no retraction events detected. Biological replicates: WT *n* = 17Δ*pilU* *n* = 16. Statistical analysis of WT vs. Δ*pilU*, unpaired two-tailed Students *t*-Test, not significant *p* = 0.1428. Source data provided as a Source Data file. **e** Representative overlay images of filtered-fluorescence and phase-contrast channels from ATPase mutants in the *mshA*^*T70C*^ strain within surface-associated cells stained with AlexaFluor 488 C_5_ maleimide dye. Images representative of three independent analyses. Scale bars = 2 μm. **f** Analysis of surface MSHA production via hemagglutination (HA) assay. The reciprocal of the lowest fold dilution at which equivalent cell levels were able to agglutinate sheep erythrocytes (HA Titer) is plotted as the mean with error bars representing the SEM. For each strain *n* = 5 biological replicates were analyzed, with two technical replicates performed for each biological replicate. ND no observable hemagglutination at the highest cell concentration. Statistical analysis: each mutant HA titer was compared to WT via unpaired two-tailed Student’s *t*-Test, ****p ≤ 0.0001. Source data provided as a Source Data file.

Upon direct microscopic visualization of MSHA pili, we were able to directly observe MSHA retraction (Fig. 2a, Supplementary Fig. 1d, and Supplementary Movies 2–4) and extension (Supplementary Fig. 1e, and Supplementary Movie 5). To validate these initial observations of retraction, we utilized a bacteriophage transduction assay with the MSHA-specific filamentous VGJΦ bacteriophage^32,33^. Upon binding to a type IV pilus receptor, filamentous bacteriophages are reportedly introduced to the cell upon pilus depolymerization/retraction^34^. Using the VGJΦ bacteriophage, tagged with a kanamycin-resistance cassette for selection of infected cells, we first confirmed that VGJΦ required MSHA for transduction. Cells lacking the major pilin subunit MshA (Δ*mshA*) showed no observable VGJΦ transduction (Fig. 2b). Addition of methoxypolyethylene glycol maleimide (MeOH-PEG-mal, ~5000 mw) to *mshA*^*T70C*^ cells, to sterically block potential MSHA retraction, resulted in a significant reduction in infected cells compared to WT treated cells (Fig. 2b). MeOH-PEG-mal interactions with the MshA^T70C^ residues, could also potentially alter VGJΦ interactions with the pilus structure. Therefore, we conclude that these results validate that VGJΦ bacteriophage transduction requires MSHA pilus production, and that blocking retraction appears to reduce transduction.

We next utilized a micropillars assay to determine the force and speed of MSHA retraction^35,36^. To ensure that micropillar readings were the result of only MSHA retraction, we generated a strain lacking the major pilin subunits of both the toxin co-regulated (TCP) and chitin-regulated competence (ChiRP) pili (Δ*tcpA* and Δ*pilA*), the major flagellum subunit (Δ*flaA*), and the second *Vibrio* polysaccharide operon (Δ*vps-*II). The average force of MSHA retraction was found to be 5.42 ± 0.78 pN (Fig. 2c) occurring at an average speed of 774 ± 205 nm per second (Fig. 2d). This force and speed are attributed specifically to MSHA pilus retraction, as there were no observable or measurable retraction events detected in the assay upon the deletion of *mshA* (Fig. 2c, d). These data further confirm that MSHA are dynamic retractile type IV pili.

### The PilT ATPase facilitates retraction of MSHA pili

We previously demonstrated that the ATPase *pilT* was necessary for MSHA-dependent biofilm formation^18^. Therefore, we hypothesized that PilT is the depolymerizing ATPase responsible for MSHA retraction. We found that MSHA production in a Δ*pilT* strain is aberrant, with reduced pilus numbers per cell (Fig. 2e), and reduced cell-surface MSHA production (Fig. 2f). However, analysis of whole-cell MshA protein levels demonstrated no significant difference for Δ*pilT* compared to WT, suggesting that loss of *pilT* does not alter pilin subunit expression (Supplementary Fig. 4). Finally, there were no observable VGJΦ transduction events, or retraction events by micropillars assay, observed in the Δ*pilT* strain (Fig. 2b–d). These data demonstrate that MSHA retraction is active via the function of PilT, and not the result of passive subunit diffusion-induced depolymerization.

MSHA pilus production in a Δ*pilU* strain was found to be equivalent to WT, with only a slight increase in VGJΦ transduction, while all phenotypes in a Δ*pilT*Δ*pilU* strain were comparable to those observed for the Δ*pilT* strain (Fig. 2b–f). Retraction force and speed measurements in the Δ*pilU* strain were equivalent to WT, at 6.09 ± 0.71 pN and 447 ± 50 nm per sec, respectively (Fig. 2c, d). Loss of the putative MSHA adhesin *mshQ*, results in a significant decrease in cell-surface MSHA production, yet pili produced are able to support VGJΦ transduction (Supplementary Fig. 5). The reduced VGJΦ transduction observed with the Δ*mshQ* strain, is likely attributed to the overall decrease in MSHA production (Supplementary Fig. 5). Therefore, we conclude that the MshQ putative adhesion is non-requisite for retraction. Together, these results demonstrate that under the conditions tested *pilT* is requisite for active MSHA retraction, and that retraction can occur independent of *pilU* and the putative adhesin MshQ.

### MSHA retraction appears common among O1 El Tor isolates

To determine if retraction was common among *V. cholerae* O1 El Tor isolates, we examined PilT-induced MSHA retraction in *V. cholerae* O1 El Tor strain E7946. Deletion of *pilT* in E7946 resulted in aberrant pilus production, with next to no observable pili on the cell surface (Supplementary Fig. 6). While MSHA production is aberrant in A1552Δ*pilT* (Fig. 2e, f), the phenotype in E7946Δ*pilT* is much more evident (Supplementary Fig. 6), highlighting strain-to-strain variations in MSHA surface dynamics among O1 El Tor isolates.

Interestingly, in E7946 attenuation of PilT ATPase function (*pilT*^K136A^) allows for proper pilus elaboration (Supplementary Fig. 6). However, loss of *pilU* or attenuation of PilU ATPase function in this background (*pilT*^*K136A*^Δ*pilU*, or *pilT*^*K136A*^*pilU*^*K134A*^) phenocopies the Δ*pilT* strain (Supplementary Fig. 6), suggesting that PilU can only function as a retraction ATPase for the MSHA system in the presence of PilT. Similar results have recently been reported for MSHA and ChiRP pili for O1 El Tor strain A1552, as well as for ChiRP in strain E7946, suggesting that this PilT-dependent PilU function is similarly conserved across O1 El Tor isolates^37,38^.

### c-di-GMP levels mediate MSHA production and retraction

The secondary messenger molecule c-di-GMP is essential for production of MSHA pili in *V. cholerae* through interactions with the polymerizing ATPase MshE^18,30,31^. Having established that the MSHA pilus possess the ability to retract, we sought to determine the impacts of c-di-GMP on both extension and retraction dynamics.

**Fig. 3:**
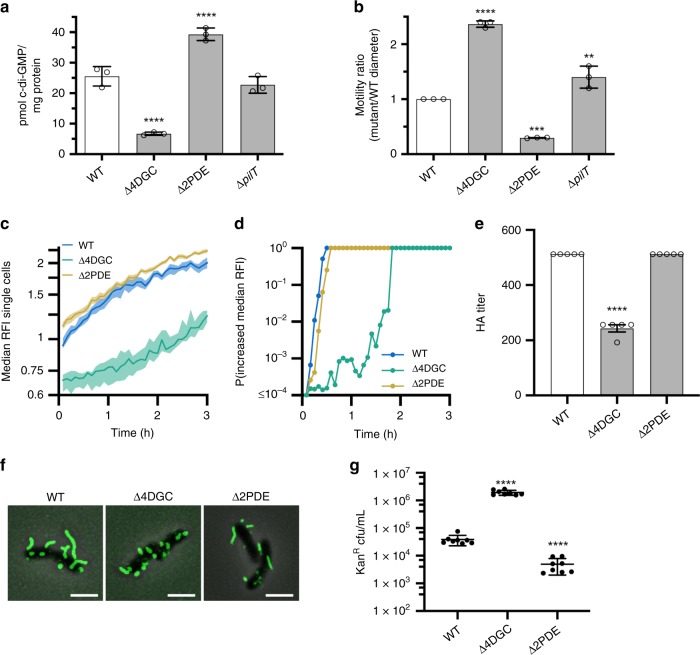
Reduced intracellular c-di-GMP levels increase flagellar motility, decrease MSHA surface piliation, and increase frequency of MSHA retraction. **a** c-di-GMP levels as quantified by LC-MS/MS. Graphs depict data as the mean, with error bars representing the standard deviation. Statistical analysis, *n* = 3 biological replicates per strain, one-way ANOVA compared to WT with Dunnett correction for multiple comparisons, ****p ≤ 0.0001. Source data provided as a Source Data file. **b** Flagellar motility in soft agar. Data presented as the ratio of mutant motility to WT motility. Graphs depict data as the mean, with error bars representing the standard deviation. *n* = 3 biological replicates per strain. Statistical analysis: one-way ANOVA compared to WT with Dunnett correction for multiple comparisons: Δ4DGC ****p ≤ 0.0001, Δ2PDE ***p = 0.0002, Δ*pilT* *p = 0.0141. Source data provided as a Source Data file. **c** Measurement of c-di-GMP levels in single cells inside a flow cell using the biosensor. Lines indicate the median RFI values per time point, and the shaded areas represent the 95% confidence intervals obtained from the bootstrap sampling distribution of the median RFI values. Time 0 H corresponds roughly to when cells initially encounter the surface of the flow cell. (**d**) Plot of the probability of c-di-GMP increase, calculated by comparing the bootstrap sampling distributions of the median RFI values between every time point (*I*_*t*_) and the first (*I*_1_). C-di-GMP is determined to be increasing if *I*_*t*_/*I*_1_ ≥ 1.05. **e** Analysis of surface MSHA production via hemagglutination (HA) assay. The reciprocal of the lowest fold dilution at which equivalent cell levels were able to agglutinate sheep erythrocytes (HA Titer) is plotted as the mean with error bars representing the SEM. For each strain *n* = 5 biological replicates were analyzed, with two technical replicates performed for each biological replicate. WT value is the same as shown in Fig. 2f. ND no observable hemagglutination at the highest cell concentration. Statistical analysis: each mutant HA titer was compared to WT via unpaired two-tailed Student’s *t*-Test, ****p ≤ 0.0001. Source data provided as a Source Data file. **f** Representative overlay images of filtered-fluorescence and phase-contrast channels of *mshA*^*T70C*^ strains within surface-associated cells stained with AlexaFluor 488 C_5_ maleimide dye. Images representative of three independent analyses. Scale bars = 2μm. **g** VGJΦ phage transduction levels. All in *mshA*^*T70C*^ strain. Individual data points of kanamycin-resistant CFU/mL plotted with line at the mean and error bars representing the standard deviation. Biological replicates: *n* = 8 per strain. Statistical analysis: unpaired two-tailed Student’s *t*-Test, ****p ≤ 0.0001. Source data provided as a Source Data file.

We have previously identified four *V. cholerae* DGCs (CdgH, CdgK, CdgL, CdgD), which promote suppression of flagellar motility through modulation of the intracellular c-di-GMP pool^39,40^. Under exponential phase planktonic growth conditions, *V. cholerae* O1 El Tor strain A1552 contains an average of 25.5 ± 3.2 pmol of c-di-GMP per milligram protein (Fig. 3a). Simultaneous deletion of these four DGCs (Δ4DGC—Δ*cdgH*Δ*cdgK*Δ*cdgL*Δ*cdgD*) significantly reduces the average c-di-GMP concentration to 6.7 ± 0.5 pmol per milligram protein, resulting in increased motility (Fig. 3a, b). To measure c-di-GMP in cells upon surface attachment, we used a c-di-GMP reporter that employs a natural in tandem repeat of c-di-GMP riboswitches controlling expression of red fluorescent protein.^41,42^ Analysis of c-di-GMP levels among WT surface-attached single cells in a flow cell model of surface attachment^26,42^, showed that intracellular c-di-GMP levels (as median reporter relative fluorescence intensity, or RFI) begin to increase immediately upon attachment to the surface (Fig. 3c), reaching a probability of the median RFI increasing of 1 within 30 min after attachment (Fig. 3d). Comparably, Δ4DGC cells show a significant lag in c-di-GMP production/accumulation (Fig. 3c), as well as significant lag in the probability of the median RFI increasing reaching 1 (Fig. 3d). Individual deletions of each DGC demonstrate similar rates of RFI increase and probabilities compared to WT (Supplementary Fig. 7a, b). This finding suggests that the lag in production/accumulation of c-di-GMP are cumulative effects of the simultaneous deletions, and not the impact of loss of any individual DGC.

Conversely, simultaneous deletion of two PDEs (Δ2PDE—Δ*rocS*Δ*cdgJ*) previously shown to decrease flagellar motility^39,43^ increases average planktonic intracellular c-di-GMP levels to 39.3 ± 2.0 pmol per milligram protein, and results in significant attenuation of motility (Fig. 3a, b). Accumulation of c-di-GMP in Δ2PDE cells upon surface attachment, mirrors that of WT cells in the rate of RFI increase and the time for the probability of the median RFI increasing to reach 1 (Fig. 3c, d). RFI values for Δ2PDE surface-attached cells are initially higher than WT, mirroring what is observed for planktonic c-di-GMP levels, and largely remain higher over the duration of the analysis (Fig. 3a, c).

We utilized Δ4DGC and Δ2PDE strains, to investigate the impact of intracellular c-di-GMP levels on MSHA production and retraction dynamics. At low c-di-GMP concentrations in the Δ4DGC strain, cell-surface MSHA production is significantly reduced compared to WT (Fig. 3e). Further analysis using MSHA labeling suggests that this reduction in MshA levels on the cell surface is the result of decreased pilus length, and not an overall impact on pilus number (Fig. 3f). Analysis of whole-cell MshA protein levels in the both the Δ4DGC and Δ2PDE strains, demonstrated no significant difference in total subunit levels compared to WT, suggesting that c-di-GMP leads to alterations in only surface MshA levels (Supplementary Fig. 4). Analysis of individual deletions of each DGC, showed no significant alterations in MSHA pilus production on the cell surface compared to WT via direct visualization (Supplementary Fig. 7c), or c-di-GMP accumulation and rate/probability of RFI increasing upon surface attachment (Supplementary Fig. 7a, b). This suggests that the effects on MSHA production in the Δ4DGC strain, likewise, results largely from changes to the overall intracellular c-di-GMP pool through cumulative loss of key DGCs. Analysis for potential c-di-GMP pathways regulating MSHA-mediated surface attachment by bacterial two-hybrid assay, also showed no interaction of the DGCs CdgH, CdgK, CdgL, or CdgD with MshE (Supplementary Fig. 8). This provides further evidence that the Δ4DGC impacts on MSHA production result from alteration of the global c-di-GMP pool, and not from MSHA-specific surface-sensing pathways.

The enhancement of planktonic c-di-GMP levels within the Δ2PDE strain demonstrated no significant alteration in cell-surface MSHA production compared to WT, and no alterations in pilus number or distribution (Fig. 3e, f). It has been previously established that intracellular c-di-GMP levels are higher in *V. cholerae* cells during exponential growth phase compared to stationary phase^44^. Analysis of reporter median RFI values upon surface attachment demonstrated lower initial c-di-GMP levels upon landing, and an increased lag period before median RFI values increase in surface-attached WT stationary phase cells compared to WT exponential phase cells (Supplementary Fig. 9a). Visualization of MSHA resulted in apparent longer pili in exponential compared to stationary phase cells, with significantly more cell-surface MSHA produced during exponential phase (Supplementary Fig. 9b–d), correlating with our observations in the Δ4DGC and Δ2PDE strains. From these data combined, we conclude that reduction in intracellular c-di-GMP levels results in decreased MSHA pilus levels on the cell surface.

We next sought to determine the impact of altered intracellular c-di-GMP levels on MSHA retraction. Analysis of VGJΦ transduction in the Δ4DGC strain, demonstrated significantly increased bacteriophage transduction compared to WT, indicative of enhanced retraction under low c-di-GMP conditions (Fig. 3g). Conversely, VGJΦ transduction levels were significantly decreased in the Δ2PDE strain compared to WT, suggesting reduced levels of retraction (Fig. 3g). VGJΦ transduction was also increased in WT stationary phase cells where c-di-GMP levels are lower, compared to exponential phase cells (Supplementary Fig. 9e). Therefore, we conclude that MSHA pilus retraction is enhanced under low intracellular c-di-GMP conditions.

### Retraction dynamics are mediated by the functional state of MshE

**Fig. 4:**
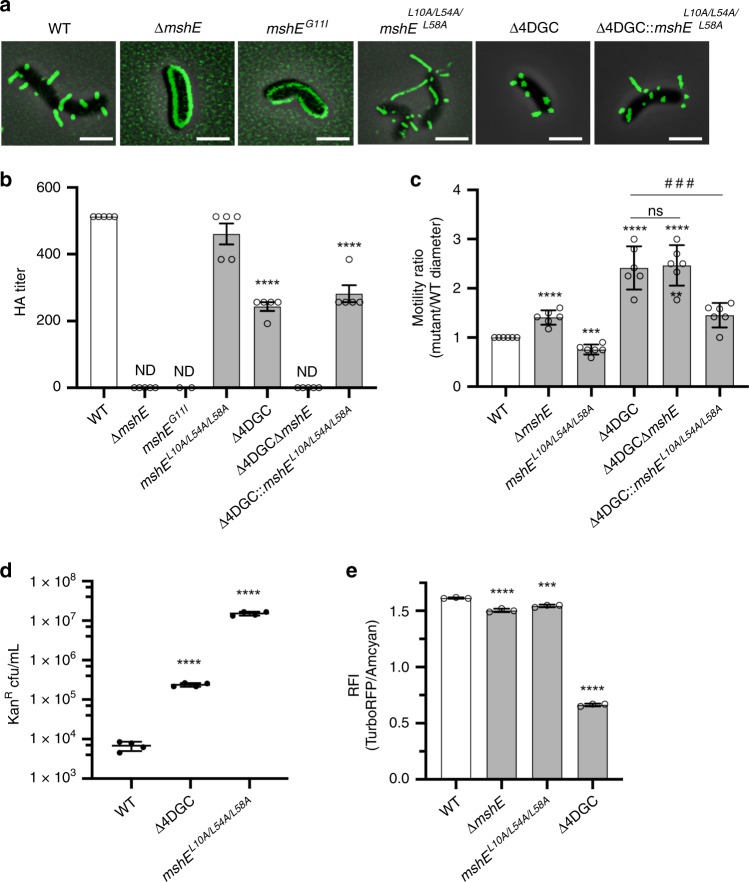
Enhanced retraction under low c-di-GMP conditions results from altered MshE activation or functional state. **a** Representative overlay images of filtered-fluorescence and phase-contrast channels of *mshA*^*T70C*^ strains within surface-associated cells stained with AlexaFluor 488 C_5_ maleimide dye. Images representative of 3 independent analyses. Scale bars = 2 μm. **b** Analysis of surface MSHA production via hemagglutination (HA) assay. The reciprocal of the lowest fold dilution at which equivalent cell levels were able to agglutinate sheep erythrocytes (HA Titer) is plotted as the mean with error bars representing the SEM. For each strain *n* = 5 biological replicates (*mshE*^*G11I*^ *n* = 2), with two technical replicates performed for each biological replicate. WT and Δ4DGC values are the same as shown in Fig. 3e. ND no observable hemagglutination at the highest cell concentration. Statistical analysis: each mutant HA titer was compared to WT via unpaired two-tailed Student’s *t*-Test, ****p ≤ 0.0001. Source data provided as a Source Data file. **c** Flagellar motility in soft agar. Data presented as the ratio of mutant motility to WT motility. Graphs depict data as the mean, with error bars representing the standard deviation. *n* = 6 biological replicates per strain. Statistical analysis: unpaired two-tailed Student’s *t*-Test, each strain compared to WT ****p ≤ 0.0001, ***p = 0.0001, **p = 0.0012; ^###^Δ4DGC vs Δ4DGC::*mshE*^*L10A/L54A/L58A*^ *p* = 0.0009, Δ4DGCΔ*mshE* vs Δ4DGC::*mshE*^*L10A/L54A/L58A*^ *p* = 0.0004. **d** VGJΦ phage transduction levels. All in *mshA*^*T70C*^ strain. Individual data points of kanamycin-resistant CFU/mL plotted with line at the mean and error bars representing the standard deviation. Biological replicates: *n* = 3 per strain. Statistical analysis: unpaired two-tailed Student’s *t*-Test, ****p ≤ 0.0001. **e** Planktonic exponential phase c-di-GMP levels determined with the Bc3-5 biosensor. Data presented as mean with standard deviation, *n* = 3 biological replicates per strain. Statistical analysis, One-way ANOVA compared to WT with Dunnett correction for multiple comparisons, ***p = 0.0002, ****p ≤ 0.0001. Source data provided as a Source Data file.

We observed that c-di-GMP promotes MSHA extension on the cell surface, suggesting enhanced MshE activity under elevated intracellular c-di-GMP conditions. Next, we sought to evaluate whether c-di-GMP-mediated effects on MSHA extension/retraction dynamics are governed through regulation of MshE activity. Deletion of *mshE*, as well as introduction of a point mutation in the c-di-GMP-binding domain (*mshE*^*G11I*^) that precludes c-di-GMP-binding, result in no observable pili on the cell-surface or MSHA-mediated hemagglutination (Fig. 4a, b). This confirms that c-di-GMP interactions with MshE are vital for extension of MSHA pili. We previously identified a series of simultaneous point mutations (*mshE*^*L10A/L54A/L58A*^) that preclude c-di-GMP-binding to MshE, yet allow for MSHA production on the cell surface (Fig. 4a). In the *mshE*^*L10A/L54A/L58A*^ strain, cell-surface MSHA levels are comparable to WT, yet by direct visual observation pili more frequently appear longer (Fig. 4a, b). Therefore, we surmise that alteration of these MshE residues, places the protein in a “constitutively active” non-c-di-GMP responsive state.

To determine if decreased MSHA production in the Δ4DGC strain results from reduced c-di-GMP-mediated MshE activity, we replaced *mshE* in the Δ4DGC strain with the “constitutively active” *mshE*^*L10A/L54A/L58A*^ variant. We then analyzed MSHA production through orthogonal assays in the Δ4DGC and Δ4DGC::*mshE*^*L10A/L54A/L58A*^ strains (Fig. 4a–c). Visually surface pili levels within the Δ4DGC::*mshE*^*L10A/L54A/L58A*^ strain were comparable to WT (Fig. 4a), and HA assays revealed cell-surface MSHA levels intermediate to Δ4DGC and WT (Fig. 4b). Flagellar motility, which exhibits an inverse relationship with MSHA production (Fig. 4c)^18^, is reduced in the Δ4DGC::*mshE*^*L10A/L54A/L58A*^ strain compared to Δ4DGC (Fig. 4c, Supplementary Fig. 10a). Collectively, these results demonstrate restoration of MSHA production within the Δ4DGC::*mshE*^*L10A/L54A/L58A*^ strain, suggesting that the reduction in cell-surface MSHA levels in the Δ4DGC strain is the result of decreased MshE extension activity. We next sought to determine the impact of the *mshE*^*L10A/L54A/L58A*^ triple point mutation on MSHA retraction. VGJΦ transduction in the *mshE*^*L10A/L54A/L58A*^ strain was elevated compared to both WT and Δ4DGC strains (Fig. 4d). Intracellular c-di-GMP levels in the *mshE*^*L10A/L54A/L58A*^ strain were similar to WT (Fig. 4e), suggesting increased VGJΦ transduction was not the result of altered c-di-GMP levels. These findings suggest that the functional state of MshE^L10A/L54A/L58A^ differs from that of c-di-GMP-activated MshE, resulting in a simultaneous increase in both MSHA extension and retraction. We therefore conclude that MSHA extension/retraction dynamics are largely governed by the functional state of MshE.

### MSHA retraction impacts near-surface motility

**Fig. 5:**
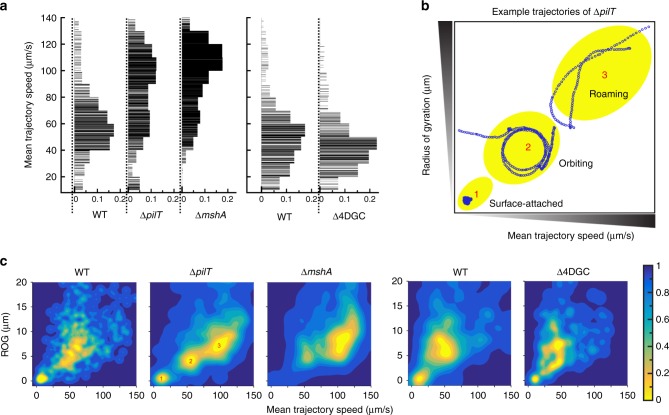
PilT-mediated MSHA retraction impacts near-surface motility patterns. **a** Comparison between the mean trajectory speed (MTS) for WT, Δ*pilT* and Δ*mshA*. A similar comparison between WT and Δ4DGC is made in a separate experiment, to minimize errors from variations of external conditions. Each horizontal line in the plot represents one trajectory. The widths of the lines represent the distribution probabilities with a bin width of 10 µm per second. **b** Representative trajectories (marked by open blue circles) from these distinctive loci (represented by underlaid yellow ovals) are shown. Locus 1 is represented with a surface-attached cells; locus 2 with an orbiting cell, and locus 3 with a roaming cell. **c** Contour maps showing the radius of gyration (ROG) vs mean trajectory speed (MTS) for all trajectories in **c**: WT, Δ*pilT* and Δ*mshA* in the first comparison, and WT and Δ4DGC in the second comparison. The contour plot for all measured trajectories are peaked at several loci: These loci can be seen most clearly in Δ*pilT*, numbered 1, 2, and 3, corresponding to the trajectories highlighted in 5b.

The MSHA pilus plays a central role in mediating near-surface motility during the initial stages of *V. cholerae* surface interactions^18^. We next sought to define the impacts of retraction on near-surface motility using high-speed microscopy. The mean trajectory speed (MTS) measures the average swimming speed for the entire trajectory of near-surface motility. Typically, cells with strong surface interactions have a low MTS, while cells with weaker or less surface interactions tend to have a high MTS. For WT, the MTS distribution exhibits a broad peak at a low MTS between 40–60 µm per second, presumably due to strong pili surface interactions (Fig. 5a). The Δ*mshA* strain exhibits a broad peak between much higher speeds of 100–120 µm per second, reflective of the low levels of interaction with the surface (Fig. 5a). In the retraction deficient Δ*pilT* strain, the MTS distribution appears to have two broad peaks; one at low speeds between 50–70 µm per second close to that of WT, and a second peak at higher speeds between 90–110 µm per second close to that of Δ*mshA* (Fig. 5a). This intermediate phenotype suggests that cells demonstrate a combination of both stronger (similar to WT) and weaker (similar to Δ*mshA*) surface interactions in the absence of *pilT* (Fig. 5a). However, the majority of Δ*pilT* interactions were weaker and more similar to Δ*mshA*, most likely due to the overall increase in the number of motile cells observed for the Δ*pilT* strain (Fig. 3b). Analysis of cell swim speeds in the bulk fluid away from the surface, demonstrated no significant differences in MTS between strains, demonstrating that MSHA retraction specifically alters near-surface motility patterns (Supplementary Fig. 10b).

The radius of gyration (ROG) measures the spatial spread of a given bacterial trajectory during near-surface motility^3^. This information can be combined with MTS measurements to highlight how mutation within the MSHA pilus machinery impacts overall near-surface motility patterns. Such analyses shown in Fig. 5b, c, are contour plots comparing the ROG to MTS for all trajectories. The contour plot for all measured trajectories shows several peaks; and these loci (yellow) are demonstrated as clearly defined peaks within the analysis of the Δ*pilT* strain and are numbered 1, 2, and 3 (Fig. 5c). Figure 5b shows representative trajectories (marked by open blue circles) from these three distinctive peaks observed for Δ*pilT*. Peak 1 shows a representative trajectory of a “surface-attached” cell with a small ROG and low MTS, peak 2 shows a representative trajectory of an “orbiting” cell exhibiting multiple near-circular tracks with intermediate ROG and MTS, and peak 3 shows a representative trajectory of a “roaming” cell with low-curvature tracks with a large ROG and high MTS (Fig. 5b), as we have previously defined^3^. Examination of the contour plot for Δ*mshA* (Fig. 5c) shows that most trajectories are similar to roaming trajectories, with no observable surface-attached cells, highlighting the low levels of surface interactions in the absence of MSHA. This data further demonstrates that MSHA are required for mediating near-surface orbiting motility and surface attachment (Fig. 5b, c), which is in agreement with our previous results^18^. The WT strain demonstrates two broad peaks within the contour plot (Fig. 5c), which are the peaks that are correspond to surface-attached and orbiting trajectories, and is missing the third peak that corresponds to roaming trajectories. Retraction deficient Δ*pilT* cells show a distribution that has characteristics of both WT and Δ*mshA* cells (Fig. 5c), with three distinct peaks representing all three categories of trajectories. Δ*pilT* cells are able to attach to the surface, and perform orbiting motilities (Fig. 5b, c), consistent with our observations that a proportion of Δ*pilT* cells retain MSHA pili (Fig. 2e, f). The proportion of surface-attached Δ*pilT* cells is reduced compared to WT, and is correlative with a sizable increase in roaming trajectories (Fig. 5c), likely attributed to decreased MSHA production (Fig. 2e, f). Surface-attached and orbiting peak trajectories are more clearly separated in the Δ*pilT* strain compared to WT, demonstrating that the Δ*pilT* strain has fewer trajectories with characteristics of both trajectories (Fig. 5c). Therefore, we conclude that MSHA pilus retraction, facilitated by PilT, aids in mediating near-surface motility.

To determine the impacts of increased retraction on near-surface motility, in separate analyses from those described above, we measured the MTS and ROG for WT and Δ4DGC strains. The MTS for WT correlated with our previous analysis (peak at 40–60 µm per second), while the MTS for Δ4DGC cells was reduced and peaked between 30–50 µm per second, suggestive of slightly stronger surface interactions compared to WT (Fig. 5a). The Δ4DGC strain contains a higher proportion of motile cells compared to WT (Figs. 3b and 4c), and has reduced cell-surface MSHA levels (Figs. 3e and 4b), suggesting that Δ4DGC cells will less frequently encounter the surface. However, near-surface motility patterns for Δ4DGC cells that do interact with the surface are similar to WT. Both WT and Δ4DGC cells demonstrate broad ROG peaks corresponding to surface-attached and orbiting trajectories (Fig. 5c), with more Δ4DGC cells exhibiting surface-attached and orbiting trajectories compared with Δ*pilT* cells (Fig. 5b, c). These data suggest that increased retraction observed with the Δ4DGC strain overall does not significantly alter near-surface motility patterns. However, compared to WT, hyper-retractile Δ4DGC cells have more trajectories with characteristics of both surface-attached and orbiting motilities compared to WT (Fig. 5b, c), thus providing further evidence to suggest that MSHA pilus retraction aids in facilitating near-surface motility and surface attachment.

### Prolonged retraction promotes detachment from the surface

**Fig. 6:**
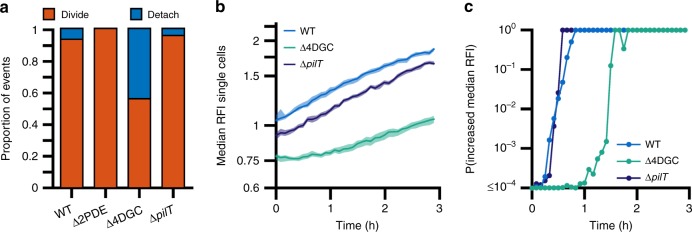
Heightened levels of MSHA retraction increases detachment from the surface, and loss of *pilT* decreases c-di-GMP levels in surface-attached cells. **a** Proportion of cells that either divide on the surface or detach from the surface. The total number of cells analyzed per strain was; WT *n* = 512, Δ2PDE *n* = 65, Δ4DGC *n* = 145, Δ*pilT* *n* = 45. The proportions are statistically significantly different among the strains according to the *χ*^2^ test *p* ≤ 0.0001. **b** Measurement of c-di-GMP levels in single cells inside a flow cell using the biosensor. Lines indicate the median RFI values per time point, and the shaded areas represent the 95% confidence intervals obtained from the bootstrap sampling distribution of the median RFI values. Time 0 h corresponds roughly to when cells initially encounter the surface of the flow cell. **c** Plot of the probability of c-di-GMP increase, calculated by comparing the bootstrap sampling distributions of the median RFI values between every time point (*I*_*t*_) and the first (*I*_1_). C-di-GMP is determined to be increasing if *I*_*t*_ / *I*_1_ ≥ 1.05.

While the Δ4DGC strain shows no significant alterations in near-surface motility patterns, it exhibits a prolonged period (~2 h) in which c-di-GMP levels fail to increase after initial attachment (Fig. 3c, d). Given this prolonged period of low c-di-GMP and heightened retraction in the Δ4DGC strain, we next utilized longitudinal family tree analysis to observe the long-term effects of prolonged duration of pilus retraction on surface attachment and colonization. This longitudinal analysis (representing a minimum time-frame of at least 3 h, and performed on the same cells used for c-di-GMP analysis) showed that 44% of Δ4DGC cells detach from surface compared to 6.8% of WT cells (Fig. 6a). This increase in detachment was correlative with decreased overall intracellular c-di-GMP levels, and a prolonged period of low probability of c-di-GMP levels increasing (Fig. 6b, c). The Δ*pilT* strain showed slightly lower levels of detachment (4.4%) compared to WT (Fig. 6a). The Δ2PDE strain which exhibits decreased MSHA retraction, showed no detachment from the surface (Fig. 6a). These data suggest that in addition to enhancing initial surface attachment, MSHA retraction also facilitates detachment from the surface. In surface-associated cells of the Δ*pilT* strain, intracellular c-di-GMP levels were significantly reduced compared to WT, but remained significantly higher than the Δ4DGC strain (Fig. 6b). The rate of c-di-GMP increase upon attachment, and the probability of c-di-GMP levels increasing, in the Δ*pilT* strain were both similar to those observed with WT (Fig. 6b, c). Collectively, these data demonstrate that proper modulation of MSHA extension and retraction activity plays a role in mediating initial surface interactions, as well as maintaining surface attachment to promote the long-term colonization necessary for biofilm formation and maturation.

### MSHA extension/retraction dynamics mediate surface colonization

**Fig. 7:**
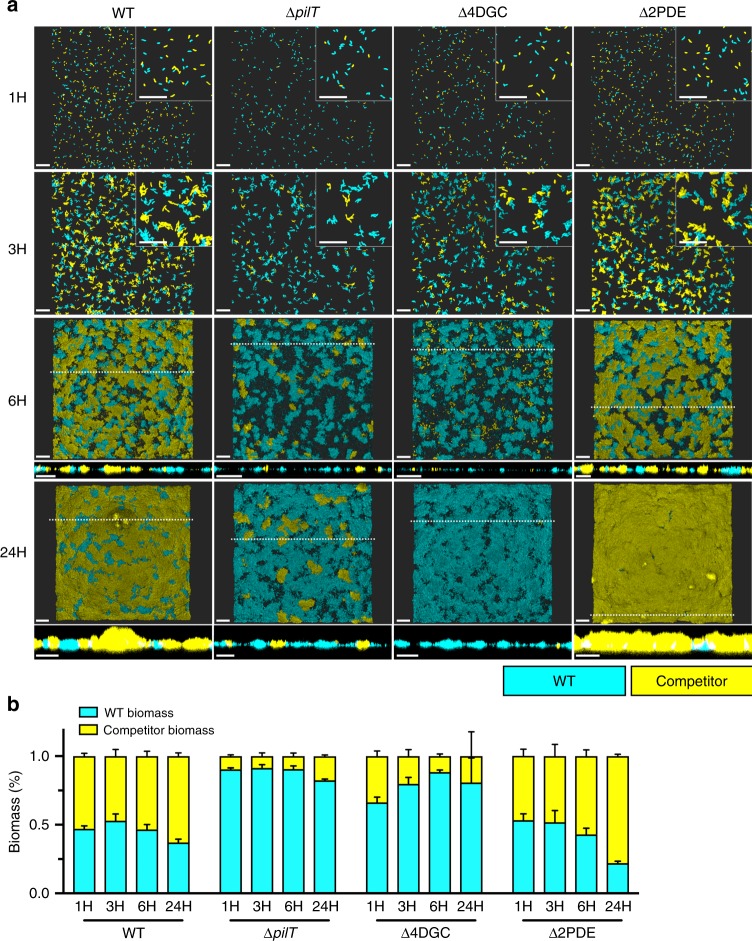
MSHA extension and retraction dynamics dictate surface attachment and biofilm production in a model of competition. **a** Representative images of flow cell biofilm competition experiments with WT::RFP (cyan) and WT::/mutant::GFP (yellow) strains, at stages corresponding to the monolayer stage (1 H), microcolony formation (3 H), three-dimensional microcolony development (6-H), and mature biofilm (24 H). Insets in the upper right corner of 1- and 3-h images are zoomed in views obtained from the same image to depict single cells and micro-colonies. Images at 6- and 24-h include a cross-sectional image of the XZ plane, and the position from which this cross-section was obtained is indicated in the image by the dashed-line. Images representative of 2 independent analyses. Scale bars = 20 μm. **b** WT::RFP (cyan) and competitor (WT::/mutant::GFP, yellow) biomass levels presented as a percentage of the overall biomass. Biomass levels determined using Comstat2, and the percentage of the GFP-biomass from the whole was determined at each time point and plotted as the mean from two biological replicates imaged at ×10 magnification (with three technical replicates per biological replicate and time point) with error bars indicating the standard error of the mean. Raw Comstat2 determined values are provided in Supplementary Table 1. Source data provided as a Source Data file.

To examine the direct physiological role of MSHA retraction in surface colonization and biofilm formation, we utilized a flow cell model of biofilm competition against a WT strain; examining time-points correlative to monolayer formation (1 H), early microcolony development (3 H), three-dimensional microcolony development (6 H), and mature biofilm formation (24 H)^18^. As a control, competition of a WT strain against another WT strain, demonstrated equivalent monolayer (representative of surface attachment ability) and downstream biofilm formation/maturation (Fig. 7, Supplementary Table 1). Strains lacking (Δ*mshA*) or strains unable to elaborate (Δ*mshE*) MSHA pili were severely attenuated for monolayer formation, and thereby downstream biofilm formation/maturation (Supplementary Fig. 11, Supplementary Table 1). The retraction deficient Δ*pilT* strain showed impaired monolayer formation compared to WT, though not as severe as Δ*mshA*, and attached cells retained the ability to form mature biofilms (Fig. 7, Supplementary Table 1). These data are consistent with our Δ*pilT* near-surface motility data, showing phenotypes intermediate to WT and Δ*mshA* strains, providing further evidence that MSHA retraction aids initial surface attachment.

The Δ4DGC strain showed only a slight impairment in monolayer formation (representing ~40% of the total biomass, Fig. 7b, Supplementary Table 1), likely attributed to the increased motility and decreased MSHA levels (Fig. 3d–f). However, following monolayer stages, Δ4DGC biomass levels rapidly decrease, representing only ~20% of the total biomass by 3 hours (Fig. 7, Supplementary Table 1). This is correlative to the increased detachment rates we observed over a similar time-frame in our longitudinal analyses (Fig. 6a). These data provide further evidence to support our hypothesis that prolonged periods of retraction during initial surface attachment promotes detachment from the surface. Conversely, the Δ2PDE strain shows monolayer formation equivalent to WT, but significantly out-competes WT at later stages of microcolony and mature biofilm formation (Fig. 7, Supplementary Table 1). Increased intracellular c-di-GMP levels within the Δ2PDE strain, are likely to activate additional c-di-GMP-regulated biofilm properties (i.e., cell signaling pathways, matrix production, etc.) resulting in the observed out-competition of WT.

We additionally analyzed biofilm invasion phenotypes, where Δ*mshA*, Δ*mshE*, Δ*pilT*, Δ4DGC, and Δ2PDE strains were tested for their ability to associate with a pre-formed mature WT biofilm (Supplementary Fig. 12). We observed that a WT invading strain can either attach to the surface of the pre-formed biofilm, or exposed abiotic surfaces as previously described^45,46^. Strains with attenuated MSHA production (Δ*mshA* and Δ*mshE*) demonstrated decreased ability to associate with the pre-formed biofilm (Supplementary Fig. 12). Similarly, the retraction deficient Δ*pilT* strain showed an intermediate phenotype to WT and Δ*mshA*. Δ4DGC and Δ2PDE strains showed decreased and increased ability to associate with the pre-formed biofilm, respectively (Supplementary Fig. 12). Collectively, these results demonstrate that proper c-di-GMP-mediated regulation of MSHA extension/retraction activity is vital for *V. cholerae* surface attachment and colonization that lends to biofilm development.

## Discussion

**Fig. 8:**
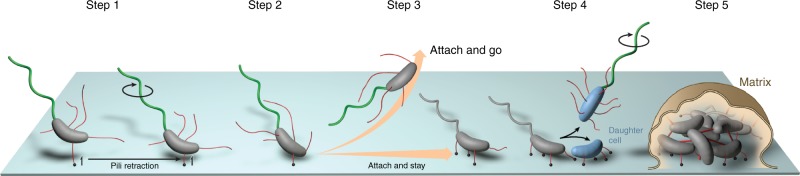
Model of MSHA-mediated initial surface-attachment dynamics of *V. cholerae*. A model through which intracellular c-di-GMP interactions with the extension ATPase MshE, modulates extension and retraction activity of the MSHA pilus to facilitate near-surface motility patterns and *V. cholerae* surface attachment. (Step 1) MSHA elaborated on the cell surface (red) facilitate initial interactions of a motile cell (gray) with the surface. Black dots in the model are indicative of MSHA interactions with the surface. As motile cells have low levels of intracellular c-di-GMP, conditions are primed to promote MSHA PilT-mediated retraction via reduced MshE extension activity. At this stage, flagellar motion (green) can continue (indicated by black circle around the flagellum) resulting in near-surface orbiting motility. (Step 2) MSHA pilus retraction aids in facilitating initial surface interactions and attachment, and then yet to be characterized intracellular signaling cascade(s) initiate an increase in intracellular c-di-GMP production that results in attenuation of flagellar-mediated motion. (Step 3) Increasing intracellular c-di-GMP levels promote MshE-mediated extension activity, to allow for further MSHA production on the cell surface and enable anchoring of the cell to the surface. Cells which do not increase intracellular c-di-GMP levels upon initial surface interactions, demonstrate extended periods of PilT-mediated retraction that can promote transient interactions and detachment from the surface. (Step 4) Upon cell division, newly produced daughter cells (blue) can either remain attached to the surface to promote microcolony formation, or return to a planktonic free-swimming state. Prolonged interactions with the surface and continual increase of intracellular c-di-GMP levels will allow for alterations in gene expression dynamics for production of components necessary for biofilm formation. (Step 5) Continued production of extracellular matrix components (matrix), and cellular replication furthers biofilm formation and maturation.

Deciphering the mechanism(s) underlying initial surface attachment is fundamental for understanding the switch from planktonic to sessile lifestyles. In this study, we define MSHA-specific initial attachment dynamics regulated by c-di-GMP in *V. cholerae*. We demonstrate that MSHA are laterally distributed retractile pili, and further confirmed PilT-mediated retraction through complementary assays. MSHA retraction was observed to drive near-surface motility patterns, and aid initial surface attachment. Moreover, we establish that the dynamics of MSHA extension and retraction are driven by the interaction of c-di-GMP with the MshE polymerization ATPase. From these data, we present a model of c-di-GMP-mediated regulation of *V. cholerae* surface attachment through direct modulation of MSHA pilus activity (Fig. 8).

Previously, we identified the MSHA polymerizing ATPase MshE as an high-affinity c-di-GMP receptor^18,30,31^. The c-di-GMP-binding motif observed in MshE is ubiquitous among many ATPases associated with type-II secretion system and type-IV pilus biogenesis^18,30,31^. While c-di-GMP is broadly conserved and is central to biofilm regulation among diverse bacterial species, our understandings of the direct molecular mechanism(s) by which c-di-GMP promotes type IV pilus production are still developing^13,23,27,28^. Here, we observe promotion of MshE extension activity at elevated c-di-GMP concentrations, resulting in a reduction of PilT-mediated retraction. This concept of direct c-di-GMP/ATPase interactions promoting type IV pilus elaboration has also been observed in *Clostridium perfringens*, where c-di-GMP-binding to the secondary PilB-homolog PilB2 promotes polymerization of PilA2^47^. Yet in other bacteria, c-di-GMP impact on type IV pilus production is mediated by different mechanisms. In *Caulobacter crescentus*, low to intermediate levels of c-di-GMP promote Tad pilus production, while high concentrations promote pilus retraction^23^. In *Pseudomonas aeruginosa*, while the PilB extension ATPase does not directly bind c-di-GMP^48^, a secondary protein FimX binds c-di-GMP to promote PilB localization to the assembly machinery and pilus elaboration^48–52^. However, FimX is only requisite for PilB localization and pilus elaboration under low c-di-GMP levels, as high intracellular c-di-GMP levels can bypass the requirement for FimX^49^.

Directionality between MshE-mediated extension or PilT-mediated retraction appears to be dependent upon the conformational state of MshE, where increased c-di-GMP-binding promotes pilus extension and represses retraction. Analysis of a c-di-GMP non-responsive “constitutively active” variant form of MshE (*mshE*^*L10A/L54A/L58A*^), demonstrated both increased pilus extension and retraction. We postulate that an altered non-optimal “active” state of MshE is responsible for the simultaneous increase in extension and retraction. Further work will be aimed at deciphering the impact of c-di-GMP-binding on the conformational or functional state of MshE, and elucidating if c-di-GMP impacts PilT-mediated retraction independent of alterations in the MshE functional state.

PilT was found to be the primary retraction ATPase facilitating MSHA depolymerization. PilT also mediates retraction of the chitin-regulated competence (ChiRP) pilus in *V. cholerae*, with a force of 10.35 ± 1.14 pN and velocity of ~100 nm per second^53^. This is the first reported instance of a single depolymerization ATPase functioning between two differential type IV pilus systems within the same bacterium. Previously in *V. cholerae*, the prepilin peptidase *pilD* (*vcpD*) has been the only factor observed to demonstrate functionality across differential systems, including the three type IV pilus (TCP, ChiRP, MSHA) and type II secretion (EPS) systems^54–57^. We determined the PilT-mediated MSHA retraction force and velocity to be 5.42 ± 0.78 pN and 774 ± 205 nm/s, respectively. Therefore, PilT depolymerization results in disparate retraction forces and velocities between the MSHA and ChiRP systems, suggesting that PilT may generate a higher retraction force when facilitating retraction at reduced velocities. These differential functions are likely key for the physiological role of each pilus system, i.e., ChiRP-mediated DNA-uptake vs. MSHA-mediated surface attachment. In other bacteria like *P. aeruginosa*, *Neisseria* gonorrheae, *Myxococcus xanthus*, and *Streptococcus sanguinis*; type IV pilus retraction has been shown to facilitate twitching motility^25,58–61^. *V. cholerae* has not been observed to exhibit twitching motility^4^, and the low force of MSHA retraction combined with lateral pilus localization are possible explanations as to why.

Analysis of the consequences of MSHA retraction in *V. cholerae* demonstrated that retraction mediates near-surface motility patterns to aid initial surface attachment. Loss of pilus retraction resulted in significant attenuation of *V. cholerae* surface colonization, while heightened retraction under prolonged reduced c-di-GMP concentrations resulted in enhanced detachment from the surface. Therefore, c-di-GMP-mediated maintenance of proper MSHA extension/retraction dynamics is likely key for mediating long-term *V. cholerae* surface attachment and downstream biofilm formation. A similar mechanism has been proposed in *C. crescentus*, where heightened c-di-GMP promotes Tad pilus retraction that not only aids in initial surface attachment, but also promotes flagellum-surface contacts that initiate production of the holdfast to facilitate long-term attachment^23^. However, even though correlations between secondary messenger signaling and pilus extension/retraction dynamics have been observed, the relationship between these signals/events are likely to be complex with both short- and long-term multigenerational temporal correlations. These correlations between secondary messenger levels (signals) and activity of motility appendages (events) can sometimes be observed in the same cell, and as well as that cell’s progeny several hours later^26^. Taken together, our results suggest that MSHA retraction serves a distinct function within *V. cholerae* surface attachment and colonization.

## Materials and methods

### Materials

Alexa Fluor 488 and 594 C5 maleimide dyes were obtained from ThermoFisher Scientific. Methoxypolyethylene glycol maleimide ~5000 mw was obtained from Sigma-Aldrich. Rabbit polyclonal anti-MshA antibody was generated from the whole protein sequence, and purified by GenScript. All animal procedures performed by GenScript in the generation of antibodies used in these studies, were performed under strict NIH standards for the care and use of animals in scientific research; as demonstrated by NIH Office of Laboratory Animal Welfare assurance #A5892-01, internal GenScript Institutional Animal Use and Care Committee guidelines, and accreditation by AAALAC International (https://www.genscript.com/NIH-grant-application-vertebrate-animal-section.html).

### Bacterial strains, plasmids, and growth conditions

Bacterial strains and plasmids used in this study are listed in Supplementary Table 2. All *V. cholerae* and *Escherichia coli* strains were grown in Luria-Bertani (LB) media at 30 °C and 37 °C, respectively, with 200 rpm shaking, unless otherwise indicated. Unless otherwise specified, all cells were grown as follows: with overnight cultures being inoculated with five single colonies from LB-agar plates into 5 mL LB media. Overnight cultures were then diluted 1:200 into either 5 or 20 mL of fresh LB, and grown to mid-exponential phase (~OD_600_ 0.5). Defined artificial seawater (DASW) media was made as previously described^62^. Flow cell biofilm cultures were performed in 2% LB media with full NaCl concentration^18^.

### A1552::*mshA*^T70C^ strain generation

The A1552 *mshA* coding region, along with 500 bp up- and downstream, were amplified from isolated genomic DNA (primers: *mshA*_500upanddown_fwd—ATCCACGAAGCTTCCCATGGGCCCCGTTTTCTTGATG, and *mshA*_500upanddown_ rev—ACTAGAGGGTACCAGAGCTCGATGCAG ACTTGGC G), and the resulting amplicon was cloned into the suicide plasmid pGP704*sacB*28. The coding sequence for threonine^70^ was then mutated to code for a cysteine, using the Q5 Site-directed Mutagenesis Kit (New England Biolabs) with the following primers (*mshA*_T70C_Fwd—AGGCATTGAATGTTTAGACTACACAGCATATACAGATG, *mshA*_T70C_Rev—TTAAT TGCAGCTCGTCCATATG). Point mutation was verified by DNA sequencing (UC Berkeley DNA Sequencing Facility), and the mutated *mshA*^*T70C*^ was then mobilized into *Vibrio cholerae* A1552 by biparental mating. The selection of double recombinants with the desired insertion of the *mshA*^*T70C*^ gene was performed as previously described^63^. To confirm insertion of *mshA*^*T70C*^, gDNA was isolated and the *mshA* coding region amplified by PCR and validated by DNA sequencing.

### Fluorescent labeling and visualization of MSHA pili

Fluorescent labeling methods were adapted from Ellison et al.^22^. Overnight cultures were inoculated with five single colonies from LB-agar plates into 5 mL LB media. Overnight cultures were diluted 1:200 into 20 mL of fresh LB, and grown to mid-exponential phase (~OD_600_ 0.5). Cells were pelleted at 6000 rpm for 5 min at room-temperature, washed with DASW, and again pelleted. Cells were then resuspended in DASW with 25–80 μg/mL Alexa Fluor^TM^ 488 or 594 C_5_ maleimide dye, and incubated at room-temperature in the dark for 15–30 min. Cells were again pelleted, washed once with DASW, and finally resuspended with LB media. Cells were then placed onto 1% agarose pads made with DASW on glass slides, cover-slipped, and sealed with valap (1:1:1 petroleum jelly/lanolin/paraffin).

Labeled cells were visualized at 63x with 1.6x auxiliary magnification on a Zeiss Axiovert 200 phase-contrast microscope outfitted with a CoolSNAP HQ2 monochrome CCD camera (Photometrics) for single image analysis, and at 100x magnification on a Zeiss AxioImager.Z2 outfitted with a Zeiss 506 monochrome camera for time-course analysis. Labeled MSHA fluorescence images from both microscopes were processed, as follows to reduce noise and strain signals and enhance MshA signals. Bandpass filtering, gamma correction, intensity percentile normalization, and then a green colormap were applied to the images. Fluorescence images were then overlaid on top of bright-field images using an opacity setting. Movies were generated with ImageJ version Fiji^64^.

### Cryo-electron tomography data collection and image analysis

*V. cholerae* strains A1552 and A1552::*mshA*^T70C^ were cultured as described above, to an OD_600_ of 0.8. Cell culture was collected, and washed once with DASW. A1552::*mshA*^T70C^ was combined with 40 μg anti-MshA antibody and incubated at room-temperature for 15 mins, while A1552 was processed without antibody addition. Cell culture was then washed twice with DASW to remove residual antibody. After mixed with 10 nm gold fiducial markers, 5 µm sample was transferred onto a freshly glow-discharged holey carbon grid (copper 200 mesh, Quantifoil) for 1 min. Blotted with a filter paper, the EM grid was rapidly plunged into liquid ethane by using a homemade plunger apparatus as previously described^65^.

EM grids with A1552 and A1552::*mshA*^T70C^ strains were transferred to a cryo-electron microscope (Titan Krios, Thermo Fisher Scientific). The microscope was equipped with field emission gun, energy filter and direct electron detector (K2, Gatan). All images were collected at focus using a Volta phase plate. Whole-cell images were collected at x26,000 magnification resulting in 0.54 nm/pixel at the specimen level. The images from cell poles were collected at x53,000 magnification resulting in 0.27 nm/pixel at the specimen level. SerialEM was used to collect cryo-ET data^66^. For better image contrast, phase shift was normally distributed in a range from 0.33π to 0.67π. A total dose of 50 e^-^/Å^2^ were distributed among 35 images covering angles from −51° to 51° at tilt steps of 3°. For every single tilt series collection, the dose-fractionated mode was used to generate 8–10 frames per projection image. Collected dose-fractionated data were first subjected to the motion correction program to generate drift-corrected tilted series^67,68^. The tilted series were aligned using gold fiducial markers by using IMOD and reconstructions were generated by using TOMO3D^69,70^. A 3D segmentation of the tomograms with whole-cell was performed by IMOD and UCSF Chimera^69,71^.

### VGJΦ bacteriophage purification and transduction assays

*Phage Purification*: *V. cholerae* strain A1552 harboring the VGJΦ bacteriophage was struck onto an LB-kanamycin (50 μg/mL) plate and incubated at 37 °C overnight. The following day, five individual colonies were inoculated into 200 mL of LB media, and incubated overnight (~18 h) at 37 °C with 225 rpm shaking. Overnight cultures were split into 4–50 mL conical vials, and centrifuged at 6000 × *g* for 30 min to pellet cells. The supernatant was then passed through a 0.45 μm filter, and then passed through a 0.20 μm filter^32,33^. An aliquot of the purified phage was plated on LB and LB-kanamycin (50 μg/mL) to check sterility of the phage stock. Working stocks were stored at 4 °C, and long-term storage was at −80 °C.

*Phage Transduction*: Overnight cultures of *V. cholerae* strains for transduction were inoculated 1:200 into 5 mL of LB media, and incubated at 30 °C with 200 rpm shaking until the OD_600_ was ~0.5. 40 μL of cells were then added to wells of a 96-well plate, followed by addition of 200 μL of purified VGJΦ phage stock. For inhibition of retraction with the mshAT70C strain, 25 μL of 50 mM MeOH-PEG-mal was added to the cells prior to the addition of the phage stock. Cells were incubated with phage for 30–60 min at room-temperature, serially diluted, and plated onto LB-kanamycin (50 μg/mL) for quantification of kanamycin-resistant colony forming units (CFUs). Plates were incubated for ~18 h at 37 °C, the number of kanamycin-resistant colonies counted, and the number of CFU/mL calculated.

### Micropillars assay

For preparation of the micropillars, silica molds were inverted on activated coverslips with polyacrylamide gels in between^35,36^. The result is an array of flexible micropillars in a hexagonal array of 3 × 3 μm with a stiffness constant of 17 ± 4 pN/μm. The gel surface was subsequently coated with 20 nm carboxylated beads to ensure adhesion of the pili. Cells from the indicated strains without any other external appendages were prepared as follows: cells were grown overnight from −80 °C stock in 3 mL of LB Miller in round-bottom polystyrene flask in a rotating incubator at 30 °C. 50 μL of the overnight suspension was added to 10 mL of LB Miller broth in an Erlenmeyer flask, and placed back in rotating incubator and monitored until the OD_600_ reached 0.7. 5–10 μL of the suspension were then added to an Attofluor experimental chamber (Thermofisher) with the pillars at the bottom, containing 500 μL of Sea Salt Solution (Instant Ocean Sea Salt 9 grams/L) and incubated in the incubator at 30 °C for ~15–30 min. The chamber was next moved to a Nikon Ti inverted microscope with an environmental chamber set at 30 °C, and 10 Hz movies of the tips of the pillars were recorded. An ImageJ plugin is then used to track the movements of the pillars with subpixel resolution, and a Matlab code allows extraction of the force and speed exerted on the pillars. All strains were imaged for a minimum of 2 h across at least three different days. Strains where no pillar movement was detected, were imaged for a minimum of 4 h.

### Hemagglutination assay

*Preparation of Sheep Erythrocytes*: Defibrinated sheep blood (Hardy Diagnostics) was used for this analysis. Erythrocytes were resuspended to a concentration of 2% in KRT buffer^72^ (10 mM Tris-HCl, pH 7.4; with 7.5 g NaCl, 0.383 g KCl, 0.318 g MgSO_4_.H_2_O, 0.305 g CaCl_2_ per liter), and pelleted at 2000 rpm for 5 min at 4 °C. Supernatant was removed, and erythrocytes were washed with KRT buffer twice or until the supernatant was clear. Erythrocytes were stored on ice when not in use.

*Assay for Hemagglutination Ability*: All strains were analyzed from planktonic exponential phase cultures (OD_600_ ~0.5–0.8), unless otherwise stated. For each strain assayed, the number of cells equivalent to an OD_600_ of 0.8 per mL were pelleted at 4000 rpm for 10 min at 4 °C. Cells were washed twice with 1 mL of KRT buffer, and finally resuspended in 100 μL of KRT buffer (for ×10 concentration of the original sample) and placed within the first well of a 96-well round-bottom plate for analysis. Fifty microliter of the cell suspension was then serially diluted down all 12 columns of the plate, and 50 μL was removed from the final column and discarded. The first row of each plate is always left blank as an untreated control. After dilution, 50 μL of the 2% sheep erythrocyte suspension was added to each well of the plate, then the plate was covered and incubated at 4 °C overnight. The last well with signs of visible hemagglutination was determined as the hemagglutination titer.

### Immunoblot analysis for whole-cell MshA subunit levels

All strains were analyzed from planktonic exponential phase cultures (OD_600_ ~0.6–8). Cells were pelleted by centrifugation, and lysed with 2% SDS in 1x PBS with protease inhibitors. Protein levels were determined by BCA Assay (Thermo Scientific), and a total of 20 μg of each sample was combined with an equal volume of 2x Laemmli sample buffer (BioRad), and loaded onto a 10-well Mini-PROTEAN TGX gel (BioRad) and run at 120 v. Once complete the gel was transferred to a nitrocellulose membrane using the Trans-Blot Turbo Transfer System (BioRad) at 1.3amp and 25 v for 7 min. Membranes were then blocked overnight 5% non-fat milk at 4 °C. Membranes were then incubated with a 1:2000 dilution of primary 1 μg/mL purified rabbit anti-MshA antibody (GenScript) for 1 h at room-temperature. Membranes were then washed 2×, and incubated with a 1:2500 dilution of 1 mg/mL HRP-conjugated goat anti-rabbit secondary antibody for 30 min at room-temperature. Membranes were then developed with SuperSignal West Pico PLUS Chemiluminescent Substrate (Thermo Scientific), and imaged on the ChemiDoc Imaging System (BioRad). Following imaging, membranes were stripped and re-blocked with 5% non-fat milk. The immunoblot analysis was then repeated with a 1:1000 dilution of 0.5 mg/mL primary purified mouse anti-*E. coli* RNA Polymerase subunit alpha antibody, and HRP-conjugated goat anti-mouse secondary antibody. For quantification, the mean pixel intensity was determined for each band using the Image Lab Software (BioRad) with analysis rectangles of the same surface area size for each band. Background was removed by using a analysis rectangle of the same surface area size from an area directly below the band analyzed. The mean pixel intensity was then determined as a percentage of the WT MshA band, and plotted.

### c-di-GMP quantification—LC-MS/MS

Intracellular c-di-GMP concentrations were quantified by LC-MS/MS as previously described^18^. Overnight cultures of each strain were diluted 1:200 in 50 mL LB media, and grown to an OD_600_ of 0.5 prior to c-di-GMP extraction.

### c-di-GMP quantification—Bc3-5 biosensor

Levels of intracellular c-di-GMP were determined with the Bc3-5 biosensor^42^. Briefly, overnight cultures in 5 mL of LB-gentamicin (15 μg/mL) were started from five single colonies struck on LB-gentamicin plates, and incubated at 30 °C with 200 rpm shaking. Cultures were diluted 1:200 into 20 mL fresh LB, without gentamicin as the plasmid harbors the Hok-Sok toxin/anti-toxin system, and incubated at 30 °C with 200 rpm shaking until the OD_600_ was ~0.5. Fluorescent intensities for amcyan and turboRFP were then determined using a Perkin-Elmer VICTOR X3 plate reader. The RFI was then determined by obtaining the ratio of turboRFP/amcyan as quantification of the amount of intracellular c-di-GMP.

### Single cell analysis flow cell experiments

*Data Acquisition*: Flow cells were prepared and inoculated as previously described^26,42^ with the following modifications: Flow cells were purchased from Ibidi (sticky-Slide *VI*^0.4^ with a glass coverslip). An in-line injection port (Ibidi) was used at the inlet for inoculating bacteria into the flow cell, and elbow connectors (Ibidi) were used to connect the chamber with tubing. Cultures for flow cells were prepared as previously described^42^ with the following modifications. The overnight culture was incubated for ~16 h before being diluted in flow cell medium (2% LB) (i.e., stationary phase culture). In addition, the overnight culture was regrown in the same overnight growth conditions to an OD_600_ ~0.6–0.8 before being diluted in flow cell medium to a final OD_600nm_ ~0.05 (i.e., exponential phase culture). The diluted bacteria culture (either stationary or exponential phase) was injected into the flow cell (which was prefilled with flow cell medium) and allowed to incubate for 10–20 min without flow on the heating stage at 30 °C. Flow was then started at 3 mL/h for the entire acquisition time. For single cell RFI measurements in flow cells, time t = 0 for each experiment correlates to when each strain has a similar number of cells in the field of view, rather than a uniform t = 0 for all strains after a certain incubation time.

Images were taken using either an Andor iXon EMCCD camera with Andor IQ software on an Olympus IX81 microscope equipped with a Zero Drift Correction autofocus system or an Andor Neo sCMOS camera with Andor IQ software on an Olympus IX83 microscope equipped with a Zero Drift Correction two continuous autofocus system^26,42^. For both systems, bright-field images were taken every 5 s (30 ms exposure time). Fluorescence images for the c-di-GMP biosensor were taken as previously described. Image size was 67 × 67 μm (1024 × 1024 pixels or 2048 × 2048 pixels). Data presented are averaged from two independent flow cell runs.

High-speed flow cell experiments were performed as previously described^42^. Empty flow cells without additional components were inoculated with the diluted bacteria culture. This flow cell protocol was also used to image the c-di-GMP biosensor fluorescence activity for Δ*pilT* strains and data related to that figure, but the imaging protocol was the same as the rest of the c-di-GMP biosensor experiments.

*Data and image analysis*: Image analysis, family tracking and manual validation, and other related calculations were performed in MATLAB^26,42^. C-di-GMP biosensor fluorescence images were processed as previously described^42^. For each fluorescence time point, the mean RFI per cell was calculated to obtain a distribution of single cell RFI values per time point. For each of these distributions per time point, the median was calculated, and then bootstrap sampling was performed to obtain a bootstrap sampling distribution of the median values. These bootstrap sampling distributions can then be used to obtain the 95% confidence intervals and directly compared to query for statistical significance.

MSHA pili count and body localization were performed from the staining fluorescence images as follows. The contour, or boundary, of the cell, obtained by binary conversion from the corresponding bright field or phase image, is transformed into a polar plot with (***r***, *θ*). The ***r*** axis points orthogonally to every point along the cell contour, with positive values corresponding to the distance away from the nearest contour point and negative values corresponding to being inside the cell contour. The *θ* axis rotates counterclockwise starting and ending from either lateral side of a cell (point along the contour that is farthest from the poles) and then normalized to be between 0 and 1. To account for an anisotropic cell shape, *θ* values increment when traversing along the perimeter of the contour rather than the actual angular values, as is the case for a circular polar plot. These values become the normalized body contour position, with values of 0 or 0.5 corresponding to either lateral side of the cell and values of 0.25 or 0.75 corresponding to either pole. Because we do not differentiate poles, these values can then be bounded between 0 and 0.25 for looking at lateral vs polar localization, or these values can be kept at the bounds between 0 and 1 (with circular wrapping) to count the number of pili around the cell. The corresponding processed fluorescence images (without the green colormap and overlay) are also transformed as described. The fluorescence intensities are averaged in the r direction to obtain an intensity vs normalized body contour position curve that peak finding is then performed on. These peaks then correspond to fluorescence spots on the original images.

### Bacterial two-hybrid assays

Bacterial two-hybrid assays between MshE and DGCs removed in the Δ4DGC background were performed as follows^73,74^. N-terminal and C-terminal translational fusions were created with DGCs of interest (CdgH, CdgK, CdgL, and CdgD), and T18 fragments of *Bordatella pertussis* adenylate cyclase (CyaA). N-terminal and C-terminal translational fusions of MshE were made with the T25 fragment of CyaA. All constructs were confirmed by DNA sequencing. Plasmids pKT25-zip and pUT18C-zip, each containing translational fusions to the leucine zipper of GCN4, were used as positive controls. Production of cAMP by reconstituted CyaA was observed in the *Escherichia coli* strains BTH101 (low c-di-GMP) or BTH101 *yhjH*::FRT (high c-di-GMP), lacking a native *cyaA* gene. Protein-protein interactions were observed by growing cells for 48 h at 30 °C on LB agar containing ampicillin (100 μg/ ml), tetracycline (15 μg/ml), X-gal (40 μg/ml), and IPTG (10–500 mM). Plates were stored overnight at 4 °C, and then imaged.

### High-speed microscopy for swimming behavior

High-speed image acquisition and analysis was performed as previously described^42^. Briefly, cells were grown in LB at 30 °C for ~18 h and then diluted 1:1000 with 2% LB (0.02% tryptone, 0.01% yeast extract, 1% NaCl, pH 7.5). The diluted cells were injected into sticky-Slide VI 0.4 flow cells (ibidi GmbH) and imaged at 200 frames per second. Cells swimming near surface are imaged at ~1 μm above surface, and cells swimming freely in the bulk solution are imaged at ~100 μm above surface. Data processing was performed using MATLAB (MathWorks). Cells that are neither swimming nor attached to the surface are excluded from analysis. The mean trajectory speed (MTS) is the average of all frame-to-frame displacement for each trajectory. The radius of gyration (ROG) measures the spread of the centroid positions for each trajectory.

### Biofilm competition assays

Overnight cultures of WT::Tn7_RFP, WT::Tn7_GFP, and Mutant::Tn7_GFP strains were inoculated into 5 mL of LB media from five single colonies, and incubated at 30 °C with 200 rpm shaking overnight (~14–18 h). WT/Mutant::Tn7_GFP strains were combined at a dilution of 1:400 individually, with a 1:400 dilution of WT::Tn7_RFP cells in 1 mL of 2% LB media, and 200 μL of the strain mixtures were pipetted into channels of an μ-Slide VI 0.4 uncoated plastic bottom slide (Ibidi) and cells were allowed to attach for 1 h at room-temperature. Following attachment, flow of 2% LB media was established at a rate of ~8 mL per channel per hour, and biofilms were allowed to form at room-temperature. Images of the developing biomass were obtained on a Zeiss LSM 880 confocal microscope at 1, 3, 6, and 24 h post establishment of flow (post-flow), at x10 magnification for biomass analysis, and 40x magnification for image generation. Images were processed with Imaris (Oxford Instruments), and biomass quantification was performed using Comstat2^75,76^.

### Flow cell biofilm invasion assays

*Biofilm Culture for Invasion*: Overnight culture of WT::Tn7_RFP was inoculated into 5 mL of LB media from five single colonies, and incubated at 30 °C with 200 rpm shaking overnight (~14–18 h). WT::Tn7_RFP was then diluted 1:200 in 1 mL of 2% LB media, and 200 μL was pipetted into all six channels of an μ-Slide VI 0.4 uncoated plastic bottom slide (Ibidi), and cells were allowed to attach for 1 h at room-temperature. Following attachment, flow of 2% LB media was established at a rate of ~8 mL per channel per hour and biofilms were allowed to form for 24 h at room-temperature.

*Biofilm Invasion*: Overnight cultures of WT::Tn7_GFP and Mutant::Tn7_GFP strains for invasion were inoculated into 5 mL of LB media from 5 single colonies, and incubated at 30 °C with 200 rpm shaking overnight (~14–18 h). Invading strains were then diluted 1:200 in 1.5 mL of 2% LB. After the WT::Tn7_RFP biofilm had been allowed to form for 24 h, invading strains were injected as follows: Injection ports (Ibidi) had been placed within the lines of the flow cell system ~30 cm upstream of the inlet port of the slide. The flow of media within the system was stopped, and the lines were clamped upstream of the injection port to prevent retrograde flow of cells upon injection. One milliliter of the 1:200 dilution of invading GFP-tagged strains was then injected through the port, using a syringe pump at a flow rate of 0.5 mL/minute. Following injection, the flow cell lines downstream of the channel were clamped, and cells were allowed to interact with the pre-formed biomass for 1 h at room-temperature. After this time, lines were unclamped and flow was re-established to the system. Images were then obtained on a Zeiss LSM 880 confocal microscope at 2- and 24-h post establishment of flow (post-flow) at ×40 magnification for image generation. Images were processed with Imaris (Oxford Instruments).

### Reporting summary

Further information on research design is available in the Nature Research Reporting Summary linked to this article.

## Supplementary information

Supplementary Information

Description of Additional Supplementary Files

Supplementary Movie 1

Supplementary Movie 2

Supplementary Movie 3

Supplementary Movie 4

Supplementary Movie 5

Reporting Summary

## Data Availability

The source data underlying Figs. 2b–d, 2f, 3a, b, 3e, 3g, 4b–e, 7b, Supplementary Figs. 1b, 4, 5b, c, 9c, e, and 11b are provided as a Source Data file. The source data for all other figures are available upon request.

## Source Data

Source Data

## References

[1] Kaper JB, Morris JG, Levine MM (1995). Cholera. Clin. Microbiol. Rev..

[2] Harris JB, Larocque RC, Qadri F, Ryan ET, Calderwood SB (2012). Cholera. Lancet.

[3] Utada AS (2014). *Vibrio cholerae* use pili and flagella synergistically to effect motility switching and conditional surface attachment. Nat. Commun..

[4] Watnick PI, Kolter R (1999). Steps in the development of a *Vibrio cholerae* El Tor biofilm. Mol. Microbiol..

[5] Moorthy S, Watnick PI (2004). Genetic evidence that the *Vibrio cholerae* monolayer is a distinct stage in biofilm development. Mol. Microbiol..

[6] Absalon C, van Dellen JG, Watnick PI (2011). A communal bacterial adhesin anchors biofilm and bystander cells to surfaces. PLoS Pathog.

[7] Berk V (2012). Molecular architecture and assembly principles of *Vibrio cholerae* biofilms. Sci. (80-.).

[8] Kierek K, Watnick PI (2003). Environmental determinants of *Vibrio cholerae* biofilm development. Appl. Environ. Microbiol..

[9] Tamayo, R., Patimalla, B. & Camilli, A. Growth in a biofilm induces a hyperinfectious phenotype in *Vibrio Cholera*. *Infect. Immun.* **78**, 3560 LP–3563569 (2010). 10.1128/IAI.00048-10 PMC2916270 20515927

[10] Faruque SM (2006). Transmissibility of cholera: in vivo-formed biofilms and their relationship to infectivity and persistence in the environment. Proc. Natl Acad. Sci. USA.

[11] Silva AJ, Benitez JA (2016). *Vibrio cholerae* biofilms and cholera pathogenesis. PLoS Negl. Trop. Dis..

[12] Colwell RR (2003). Reduction of cholera in Bangladeshi villages by simple filtration. Proc. Natl Acad. Sci..

[13] Conner JG, Teschler JK, Jones CJ, Yildiz FH (2016). Staying Alive: *Vibrio cholerae*’s cycle of environmental survival, transmission, and dissemination. Microbiol. Spectr..

[14] Marsh JW, Sun D, Taylor RK (1996). Physical linkage of the *Vibrio cholerae* mannose-sensitive hemagglutinin secretory and structural subunit gene loci: identification of the mshG coding sequence. Infect. Immun..

[15] Chiavelli DA, Marsh JW, Taylor RK (2001). The mannose-sensitive hemagglutinin of *Vibrio cholerae* promotes adherence to zooplankton. Appl. Environ. Microbiol..

[16] Jonson G, Holmgren J, Svennerholm A-M (1991). Identification of a mannose-binding pilus on *Vibrio cholerae* El Tor. Microb. Pathog..

[17] Marsh JW, Taylor RK (1999). Genetic and transcriptional analyses of the *Vibrio cholerae* mannose-sensitive hemagglutinin type 4 pilus gene locus. J. Bacteriol..

[18] Jones CJ (2015). C-di-GMP regulates motile to sessile transition by modulating MshA Pili biogenesis and near-surface motility behavior in *Vibrio cholerae*. PLOS Pathog..

[19] Watnick PI, Fullner KJ, Kolter R (1999). A role for the mannose-sensitive hemagglutinin in biofilm formation by *Vibrio cholerae* El Tor. J. Bacteriol..

[20] Melville S, Craig L (2013). Type IV pili in Gram-positive bacteria. Microbiol. Mol. Biol. Rev..

[21] Hospenthal MK, Costa TRD, Waksman G (2017). A comprehensive guide to pilus biogenesis in Gram-negative bacteria. Nat. Rev. Microbiol..

[22] Ellison CK (2017). Obstruction of pilus retraction stimulates bacterial surface sensing. Sci. (80-.).

[23] Sangermani M, Hug I, Sauter N, Pfohl T, Jenal U (2019). Tad pili play a dynamic role in *Caulobacter crescentus*; surface colonization. MBio.

[24] Maier B, Wong GCL (2015). How bacteria use type IV pili machinery on surfaces. Trends Microbiol..

[25] Skerker JM, Berg HC (2001). Direct observation of extension and retraction of type IV pili. Proc. Natl Acad. Sci. USA.

[26] Lee CK (2018). Multigenerational memory and adaptive adhesion in early bacterial biofilm communities. Proc. Natl Acad. Sci. USA.

[27] Hengge R (2009). Principles of c-di-GMP signalling in bacteria. Nat. Rev. Microbiol..

[28] Römling U, Galperin MY, Gomelsky M (2013). Cyclic di-GMP: the first 25 years of a universal bacterial second messenger. Microbiol. Mol. Biol. Rev..

[29] Sondermann H, Shikuma NJ, Yildiz FH (2012). You’ve come a long way: C-di-GMP signaling. Curr. Opin. Microbiol..

[30] Roelofs KG (2015). Systematic identification of cyclic-di-GMP binding proteins in *Vibrio cholerae* reveals a novel class of cyclic-di-GMP-binding ATPases associated with type II secretion systems. PLoS Pathog..

[31] Wang Y-C (2016). Nucleotide binding by the widespread high-affinity cyclic di-GMP receptor MshEN domain. Nat. Commun..

[32] Campos J (2003). VGJφ, a novel filamentous phage of *Vibrio cholerae*, integrates into the same chromosomal site as CTXφ. J. Bacteriol..

[33] Campos J (2003). Novel type of specialized transduction for CTXφ or its satellite phage RS1 mediated by filamentous phage VGJφ in *Vibrio cholerae*. J. Bacteriol..

[34] Rakonjac J, Bennett NJ, Spagnuolo J, Gagic D, Russel M (2011). Filamentous bacteriophage: biology, phage display and nanotechnology applications. Curr. Issues Mol. Biol..

[35] Biais N, Ladoux B, Higashi D, So M, Sheetz M (2008). Cooperative retraction of bundled type IV pili enables nanonewton force generation. PLoS Biol..

[36] Biais, N., Higashi, D., So, M. & Ladoux, B. in *Neisseria meningitidis: Advanced Methods and Protocols* (ed. Christodoulides, M.) 197–216 (Humana Press, 2012).

[37] Chlebek JL (2019). PilT and PilU are homohexameric ATPases that coordinate to retract type IVa pili. PLoS Genet..

[38] Adams DW, Pereira JM, Stoudmann C, Stutzmann S, Blokesch M (2019). The type IV pilus protein PilU functions as a PilT-dependent retraction ATPase. PLoS Genet.

[39] Liu X, Beyhan S, Lim B, Linington RG, Yildiz FH (2010). Identification and characterization of a phosphodiesterase that inversely regulates motility and biofilm formation in *Vibrio cholerae*. J. Bacteriol..

[40] Townsley L, Yildiz FH (2015). Temperature affects c-di-GMP signalling and biofilm formation in *Vibrio cholerae*. Environ. Microbiol..

[41] Zhou H (2016). Characterization of a natural triple-tandem c-di-GMP riboswitch and application of the riboswitch-based dual-fluorescence reporter. Sci. Rep..

[42] Zamorano-Sánchez D (2019). Functional specialization in *Vibrio cholerae* diguanylate cyclases: distinct modes of motility suppression and c-di-GMP production. MBio.

[43] Lim B, Beyhan S, Meir J, Yildiz FH (2006). Cyclic-diGMP signal transduction systems in *Vibrio cholerae*: Modulation of rugosity and biofilm formation. Mol. Microbiol..

[44] Koestler BJ, Waters CM (2014). Bile acids and bicarbonate inversely regulate intracellular cyclic di-GMP in *Vibrio cholerae*. Infect. Immun..

[45] Nadell CD, Drescher K, Wingreen NS, Bassler BL (2015). Extracellular matrix structure governs invasion resistance in bacterial biofilms. ISME J..

[46] Nadell CD, Ricaurte D, Yan J, Drescher K, Bassler BL (2017). Flow environment and matrix structure interact to determine spatial competition in *Pseudomonas aeruginosa* biofilms. elife.

[47] Hendrick WA, Orr MW, Murray SR, Lee VT, Melville SB (2017). Cyclic di-GMP binding by an assembly ATPase (PilB2) and control of type IV pilin polymerization in the Gram-positive pathogen *Clostridium perfringens*. J. Bacteriol..

[48] Guzzo CR, Salinas RK, Andrade MO, Farah CS (2009). PILZ protein structure and interactions with PILB and the FIMX EAL domain: implications for control of type IV pilus biogenesis. J. Mol. Biol..

[49] Jain R, Sliusarenko O, Kazmierczak BI (2017). Interaction of the cyclic-di-GMP binding protein FimX and the Type 4 pilus assembly ATPase promotes pilus assembly. PLoS Pathog..

[50] Huang B, Whitchurch CB, Mattick JS (2003). FimX, a multidomain protein connecting environmental signals to twitching motility in *Pseudomonas aeruginosa*. J. Bacteriol..

[51] Kazmierczak BI, Lebron MB, Murray TS (2006). Analysis of FimX, a phosphodiesterase that governs twitching motility in *Pseudomonas aeruginosa*. Mol. Microbiol..

[52] Qi Y (2012). Functional divergence of FimX in PilZ binding and type IV Pilus regulation. J. Bacteriol..

[53] Ellison CK (2018). Retraction of DNA-bound type IV competence pili initiates DNA uptake during natural transformation in *Vibrio cholerae*. Nat. Microbiol..

[54] Marsh JW, Taylor RK (1998). Identification of the *Vibrio cholerae* type 4 prepilin peptidase required for cholera toxin secretion and pilus formation. Mol. Microbiol..

[55] Fullner KJ, Mekalanos JJ (1999). Genetic characterization of a new type IV-A pilus gene cluster found in both classical and El Tor biotypes of *Vibrio cholerae*. Infect. Immun..

[56] Scott ME, Dossani ZY, Sandkvist M (2001). Directed polar secretion of protease from single cells of *Vibrio cholerae* via the type II secretion pathway. Proc. Natl Acad. Sci. USA.

[57] Seitz P, Blokesch M (2013). DNA-uptake machinery of naturally competent *Vibrio cholerae*. Proc. Natl Acad. Sci. USA.

[58] Gurung I (2016). Functional analysis of an unusual type IV pilus in the Gram-positive *Streptococcus sanguinis*. Mol. Microbiol..

[59] Jakovljevic V, Leonardy S, Hoppert M, Søgaard-Andersen L (2008). PilB and PilT are ATPases acting antagonistically in type IV pilus function in *Myxococcus xanthus*. J. Bacteriol..

[60] Bischof LF, Friedrich C, Harms A, Søgaard-Andersen L, Van Der Does C (2016). The Type IV pilus assembly ATPase PilB of *Myxococcus xanthus* interacts with the inner membrane platform protein PilC and the nucleotide-binding protein PilM. J. Biol. Chem..

[61] Clausen M, Jakovljevic V, Søgaard-Andersen L, Maier B (2009). High-force generation is a conserved property of type IV pilus systems. J. Bacteriol..

[62] Meibom KL, Blokesch M, Dolganov NA, Wu C-Y, Schoolnik GK (2005). Chitin induces natural competence in *Vibrio cholerae*. Science.

[63] Fong JC (2017). Structural dynamics of RbmA governs plasticity of *Vibrio cholerae* biofilms. elife.

[64] Schindelin J (2012). Fiji: An open-source platform for biological-image analysis. Nat. Methods.

[65] Zhu, S., Qin, Z., Wang, J., Morado, D. R. & Liu, J. in *The Bacterial Flagellum:* Methods *and Protocols* (eds. Minamino, T. & Namba, K.) 229–242 (Springer New York, 2017).

[66] Mastronarde DN (2005). Automated electron microscope tomography using robust prediction of specimen movements. J. Struct. Biol..

[67] Li X (2013). Electron counting and beam-induced motion correction enable near-atomic-resolution single-particle cryo-EM. Nat. Methods.

[68] Morado, D. R., Hu, B. & Liu, J. Using tomoauto: a protocol for high-throughput automated cryo-electron tomography. *J. Vis. Exp*. **107**, 1–11 (2016). 10.3791/53608 PMC4781705 26863591

[69] Kremer JR, Mastronarde DN, McIntosh JR (1996). Computer visualization of three-dimensional image data using IMOD. J. Struct. Biol..

[70] Agulleiro JI, Fernandez JJ (2015). Tomo3D 2.0 - exploitation of advanced vector eXtensions (AVX) for 3D reconstruction. J. Struct. Biol..

[71] Pettersen EF (2004). UCSF Chimera—a visualization system for exploratory research and analysis. J. Comput. Chem..

[72] Gardel CL, Mekalanos JJ (1996). Alterations in *Vibrio cholerae* motility phenotypes correlate with changes in virulence factor expression. Infect. Immun..

[73] Karimova G, Pidoux J, Ullmann A, Ladant D (1998). A bacterial two-hybrid system based on a reconstituted signal transduction pathway. Proc. Natl Acad. Sci. USA.

[74] Shikuma NJ, Fong JCN, Yildiz FH (2012). Cellular levels and binding of c-di-GMP control subcellular localization and activity of the *Vibrio cholerae* transcriptional regulator VpsT. PLoS Pathog.

[75] Givskov M (2015). Quantification of biofilm structures by the novel computer program comstat. Microbiology.

[76] Vorregaard, M. Comstat2 - a modern 3D image analysis environment for biofilms. *Informatics and Mathematical Modelling*, (Technical University of Denmark, Denmark, 2008).

