# Supplementary material for: c-di-GMP modulates type IV MSHA pilus retraction and surface attachment in Vibrio cholerae
Source: Nat Commun. 2020 Mar 25;11:1549. doi: 10.1038/s41467-020-15331-8 (PMC7096442; doi:10.1038/s41467-020-15331-8)
Supplement: Supplementary file 1 — Supplementary Information [file 41467_2020_15331_MOESM1_ESM.pdf]

Supplementary Materials for:

c-di-GMP modulates type IV MSHA pilus retraction and surface attachment in *Vibrio cholerae*

Kyle A. Floyd[1], Calvin K. Lee[2], Wujing Xian[2], Mahmoud Nametalla[3,4], Aneesa Valentine[3,4], Benjamin Crair[5], Shiwei Zhu[5], Hannah Q. Hughes[6], Jennifer L. Chlebek[6], Daniel C. Wu[1], Jin Hwan Park[1], Ali M. Farhat[2], Charles J. Lomba[2], Courtney K. Ellison[6,$], Yves V. Brun[7], Javier Campos-Gomez[8], Ankur B. Dalia[6], Jun Liu[5], Nicolas Biais[3,4], Gerard C. L. Wong[2,*], Fitnat H. Yildiz[1,*]

[1]Department of Microbiology and Environmental Toxicology, University of California – Santa Cruz, 1156 High St., BioMed 245, Santa Cruz, California, 95064, USA

[2]Departments of Bioengineering, Chemistry and Biochemistry, California Nano Systems Institute, University of California – Los Angeles, 420 Westwood Plaza, Room 5121 Engineering V, Los Angeles, California, 90095, USA

[3]Department of Biology, Brooklyn College, Room 307NE, 2900 Bedford Ave., Brooklyn, New York, 11210, USA

[4]CUNY Graduate Center, 365 5th Ave., New York, New York, 10016, USA

[5]Department of Microbial Pathogenesis, Yale University, 840 West Campus Drive, Advanced Biosciences Center 211, West Haven, CT, 06516, USA

[6]Department of Biology, Indiana University – Bloomington, 1001 East Third St., Jordan Hall 469A, Bloomington, Indiana, 47405, USA

[7]Department of Microbiology, Infectious Diseases, and Immunology, Faculty of Medicine, University of Montreal, Pavillon Roger-Gaudry, 2900, boulevard Édouard-Montpetit, C.P. 6128, Succursale Centre-ville, Montréal, QC H3C 3J7.

[8]Cystic Fibrosis Research Center, University of Alabama at Birmingham, 1918 University Blvd., MCLM 702, Birmingham, AL, 35233, USA

[$]Current Affiliation: Lewis-Sigler Institute for Integrative Genomics, Princeton University, 355 Thomas Laboratory, Washington Road, Princeton, NJ, 08544, USA.

*Co-corresponding authors: gclwong@seas.ucla.edu and fyildiz@ucsc.edu

This PDF file includes:

- Supplementary Figures 1 – 12
- Supplementary Tables 1 – 2
- Supplementary References

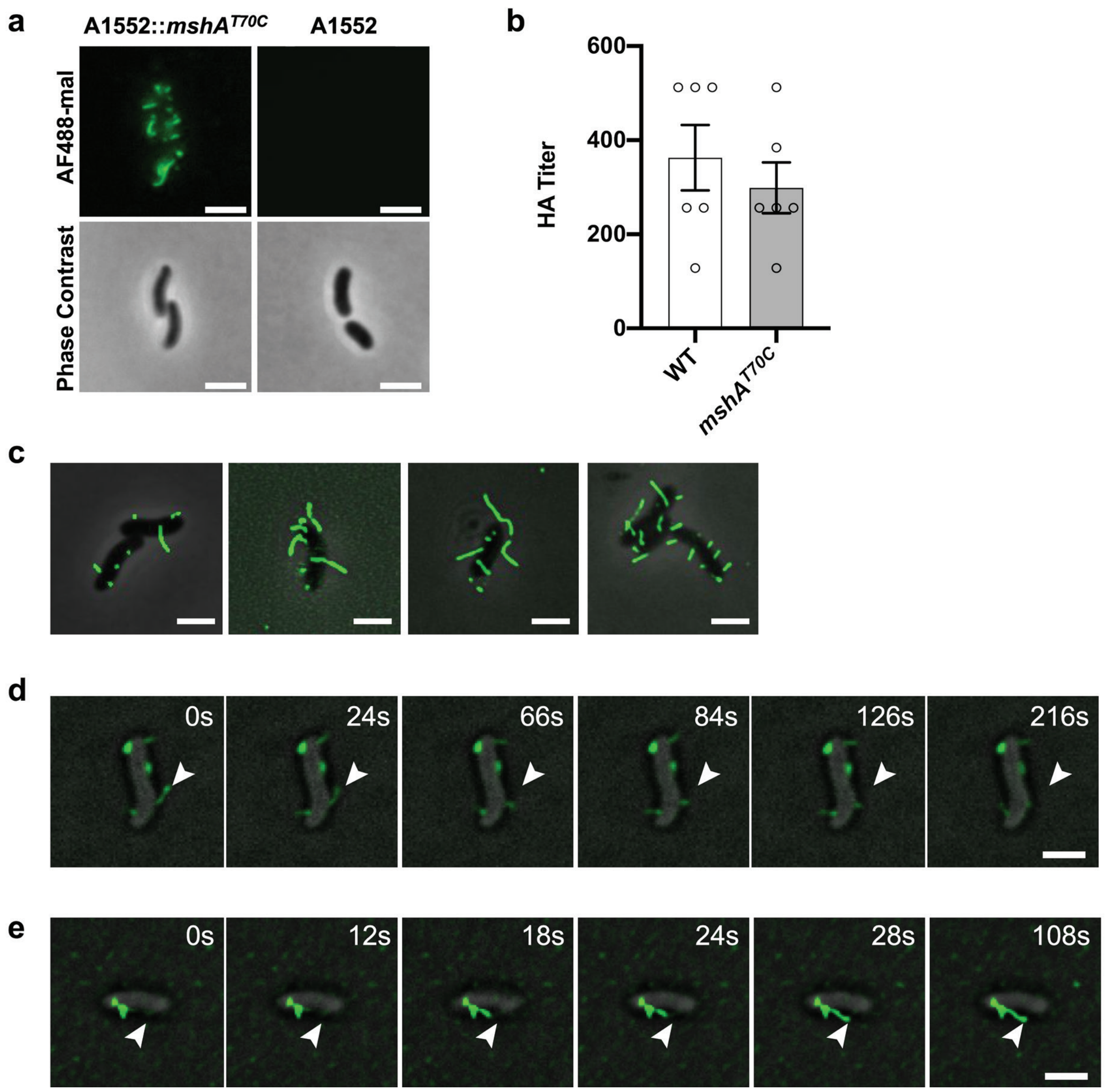

Supplementary Figure 1. Fluorescent microscopy visualization of MSHA pili.
(**a**) Labeling of the *mshA*^{T70C} strain is distinct and specific, as a WT non-*mshA*^{T70C} strain shows no labeling with the maleimide dye. Images of fluorescence and phase-contrast channels for WT and *mshA*^{T70C} surface-associated cells, representative of 3 independent analyses. Scale bar = 2 μm.
(**b**) Analysis of surface MSHA production via hemagglutination (HA) assay. Reciprocal of the HA Titer is plotted as mean with error bars representing the SEM. Each strain n = 6 biological replicates were analyzed, with two technical replicates per biological replicate. Statistical analysis: mutant compared to WT via unpaired two-tailed Student's *t*-Test, $p = 0.4835$. Source data provided as a Source Data file.
(**c**) Overlay images of filtered-fluorescence and phase-contrast channels of labeled MSHA pili on surface-associated *mshA*^{T70C} cells. Representative of 3 independent analyses. Scale bar = 2 μm.
(**d**) Representative overlay images of filtered-fluorescence and phase-contrast channels demonstrating pilus retraction in surface-associated *mshA*^{T70C} cells. Images collected every 6 seconds for 10 minutes. White arrow in all images marks the tip of the retracting pilus at t = 0. Numerous retraction events were observed over multiple biological replicates (see Supplementary Movies). Scale bar = 2 μm.
(**e**) Representative overlay images of filtered-fluorescence and phase-contrast channels demonstrating pilus extension in surface-associated *mshA*^{T70C} cells. Images were collected every 6 seconds for 6 minutes. White arrow in all images marks the position at which the tip of the extending pilus will be positioned at t = 108s. Numerous extension events were observed over multiple biological replicates. Scale bar = 2 μm.

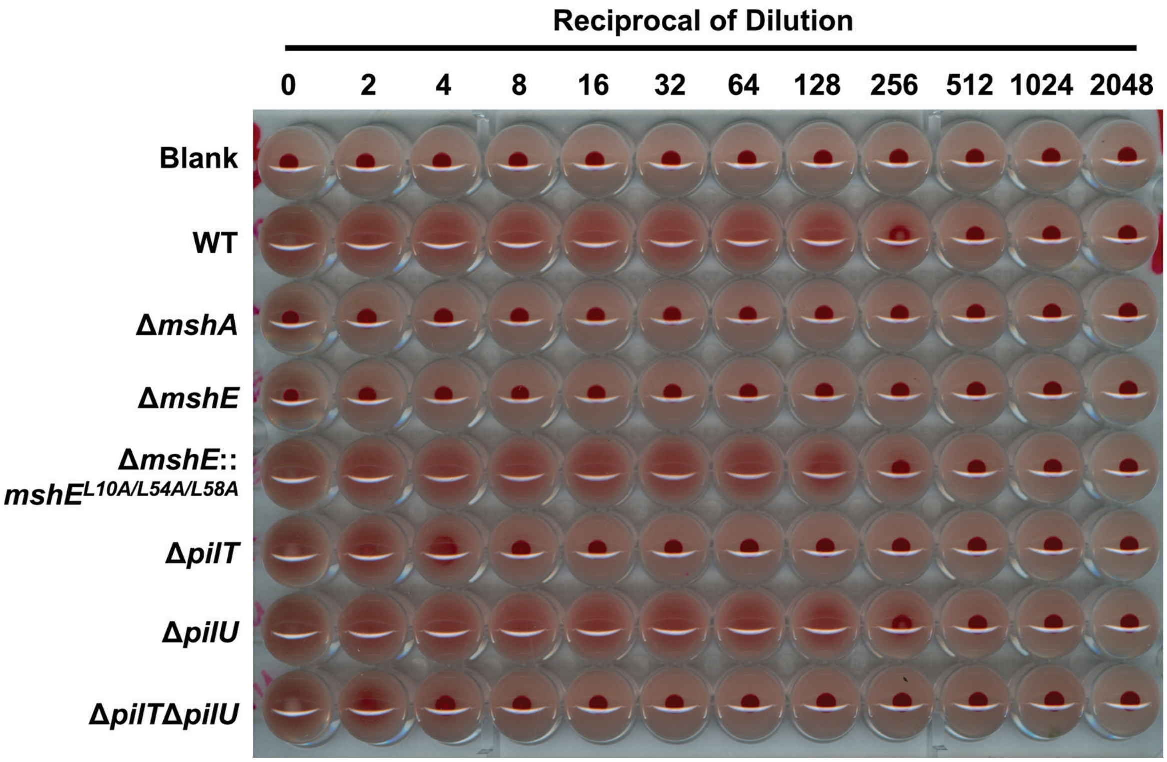

Supplementary Figure 2. Representative output of MSHA-specific hemagglutination (HA) assay. Hemagglutination is defined as the ability of bacteria to keep sheep erythrocytes in suspension, and from pelleting at the bottom of the well. Using this HA assay with sheep erythrocytes demonstrates MSHA-specific hemagglutination, as cells lacking the major pilin subunit (Δ*mshA*) or unable to produce MSHA pili (Δ*mshE*) demonstrate no hemagglutination ability. The HA titer is defined as the reciprocal of the highest dilution at which the strain is able to hemagglutinate. Therefore, strains with reduced cell surface MSHA levels will hemagglutinate at lower dilutions compared to cells with higher surface MSHA levels.

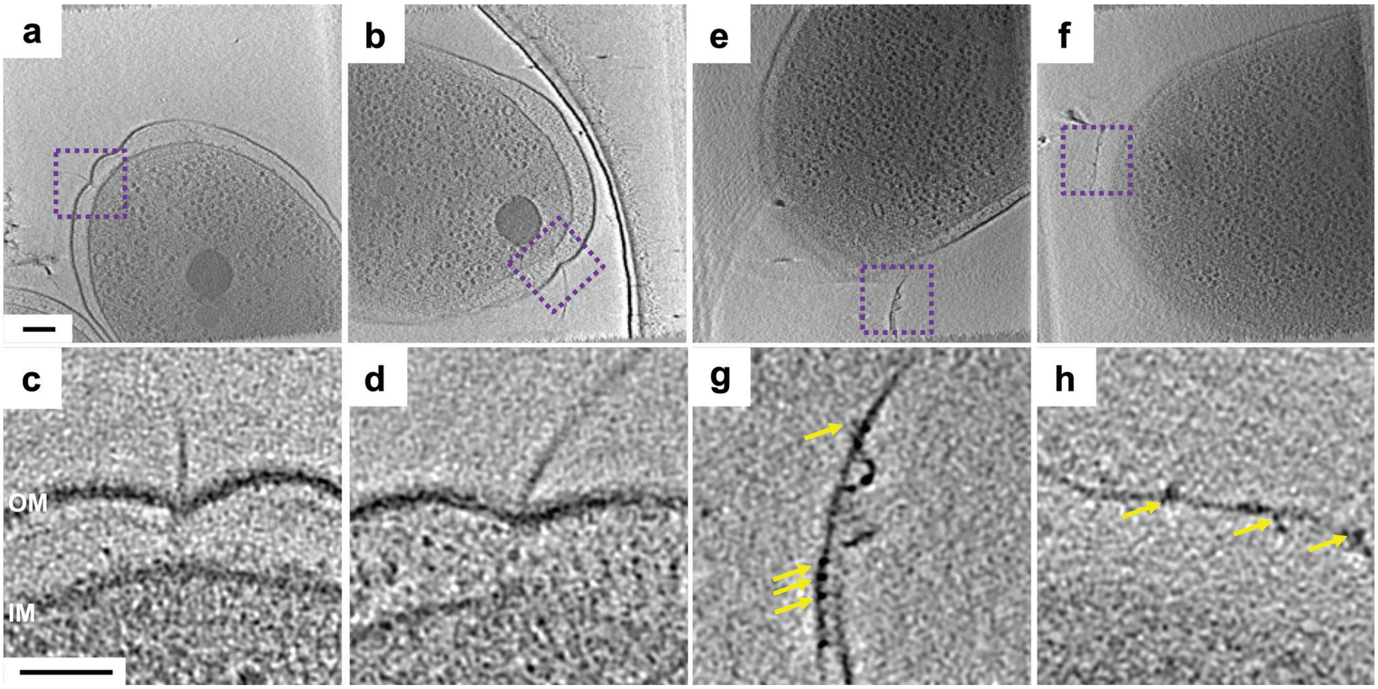

Supplementary Figure 3. Cryo-ET imaging of cell-surface MSHA pili at 53,000x magnification with and without anti-MshA antibody.

(**a, b**) Representative slices of tomograms from two individual A1552 cells in absence of antibody.

(**c, d**) Zoom-in images of pilus structures as boxed in purple in **a** and **b**.

(**e, f**) Representative slices of tomograms from two individual A1552::*mshA*^{T70C} cells treated with anti-MshA antibody.

(**g, h**) Zoom-in images of pilus structures as boxed in purple in **e** and **f**. Yellow arrows indicate anti-MshA antibody coated on the surface of MSHA pili. OM, outer membrane. IM, inner membrane. Scale bar in top panels is 100 nm, and 50 nm in bottom panels. Pixel size is 0.55 nm.

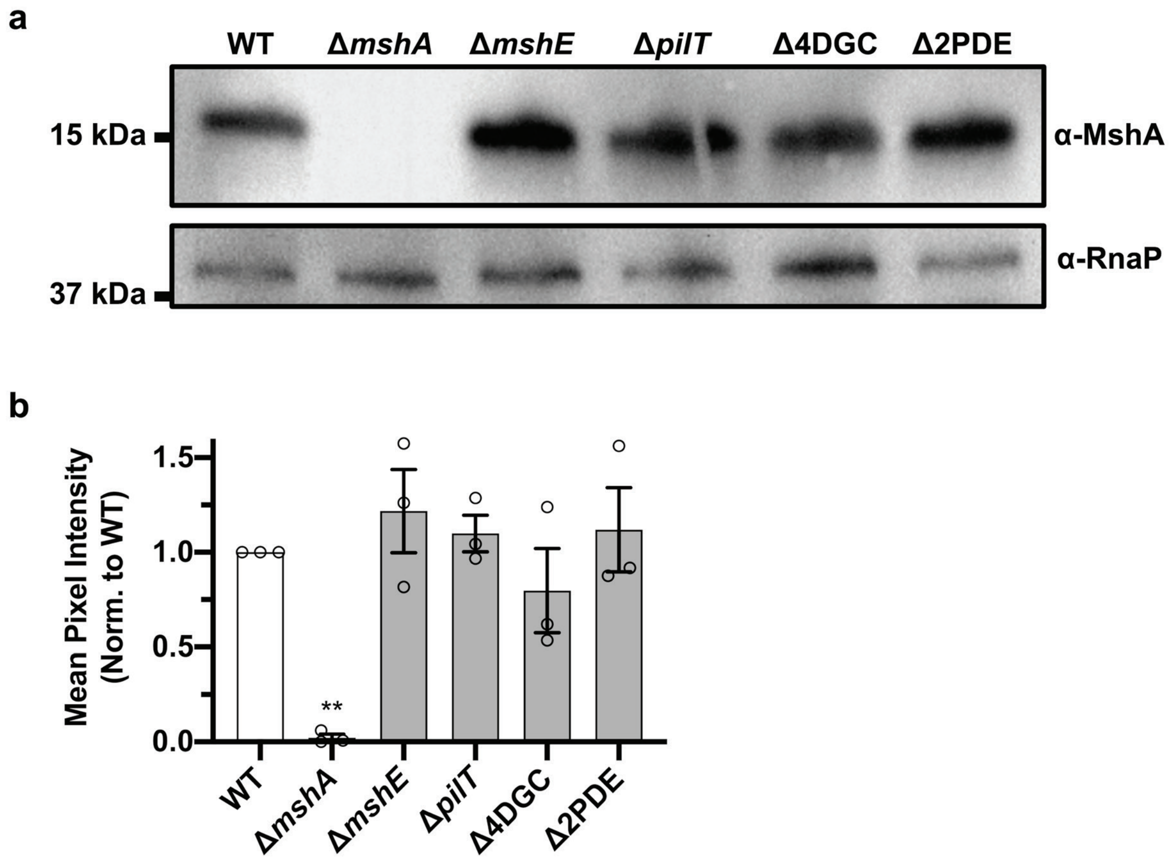

Supplementary Figure 4. Loss of ATPases or alterations in intracellular c-di-GMP levels do not significantly alter whole cell MshA protein levels.

(**a**) Representative image of anti-MshA immunoblot for whole cell MshA protein levels. Image is representative of 3 total immunoblots, each loaded with 20 μg of protein for each sample. After blotting for MshA, the same membrane was stripped and re-blotted for RNA polymerase alpha subunit as a loading control. Source data provided as a Source Data file.

(**b**) Mean pixel intensity (MPI) as a percentage of WT for MshA bands. Data presented as the mean with the SEM from three immunoblots of three separate biological replicates. MPI was determined using the BioRad Image Lab Software. An analysis rectangle was drawn around the band to determine the MPI, and the exact same size analysis rectangle was used for each band. Background levels were determined from just below the band analyzed in each lane. MPI was then converted to a percentage of the WT to correct for variations in antibody interactions across different blots. Statistical analysis, One-way ANOVA compared to WT with Dunnett correction for multiple comparisons, $^{**}p = 0.0044$. Source data provided as a Source Data file.

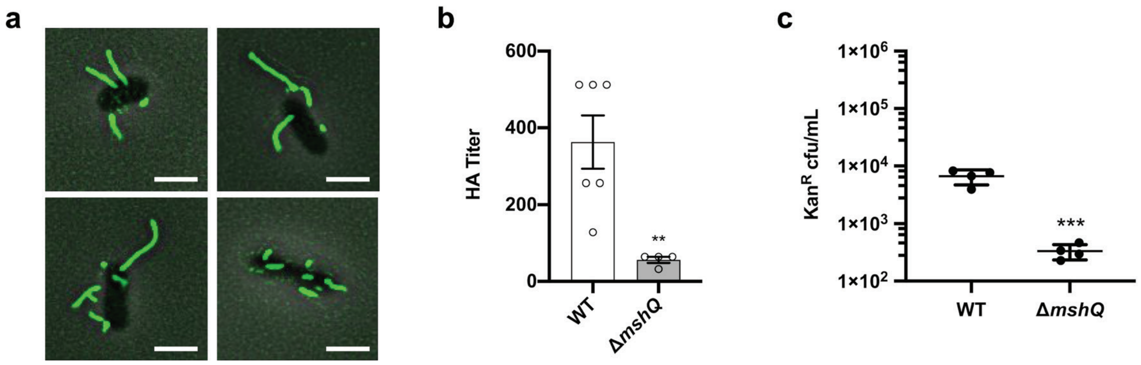

Supplementary Figure 5. The MSHA putative adhesin, MshQ, is non-essential for pilus retraction.
(**a**) Representative overlay images of filtered-fluorescence and phase-contrast channels depicting labeled MSHA pili from the surface-associated Δ*mshQ* strain. Images representative of 3 independent analyses. Scale bar = 2 μm.
(**b**) Analysis of surface MSHA production via hemagglutination (HA) assay. The reciprocal of the lowest fold dilution at which equivalent cell levels were able to agglutinate sheep erythrocytes (HA Titer) is plotted as the mean with error bars representing the SEM. WT n = 4 and Δ*mshQ* n = 6 biological replicates were analyzed, with two technical replicates performed for each biological replicate. WT value is the same as shown in Supplementary Figure 1b. Statistical analysis: mutant HA titer compared to WT via unpaired two-tailed Student's *t*-Test, ***p* = < 0.0078. Source data provided as a Source Data file.
(**c**) VGJΦ phage transduction levels. Individual data points of kanamycin-resistant CFU/mL plotted with line at the mean and error bars representing the standard deviation. Biological replicates: WT n = 4, Δ*mshQ* n = 4. Statistical analysis: unpaired two-tailed Student's *t*-Test, ****p* = 0.0001. Source data provided as a Source Data file.

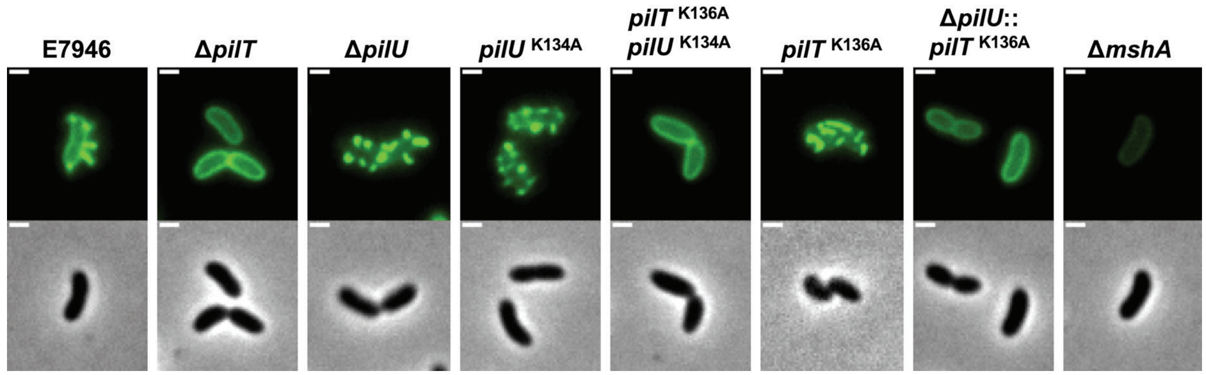

Supplementary Figure 6. PilT is essential for MSHA retraction in *V. cholerae* isolate E7946, while PilU is sufficient but not requisite.
Representative fluorescent and phase contrast images of the indicated strains where pili are labeled with AF488-mal. Images representative of 3 independent analyses. Scale bar = 1 μm.

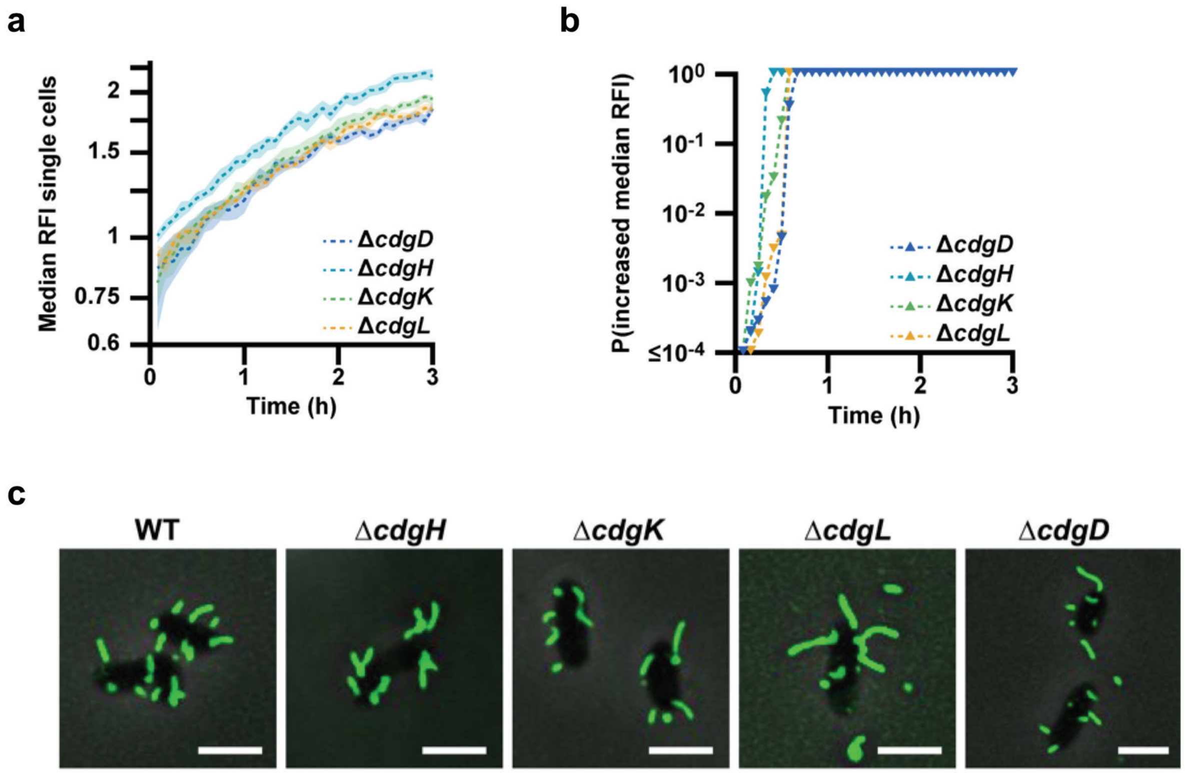

Supplementary Figure 7. Individual deletion of DGCs within the Δ4DGC strain does not impact MSHA production or intracellular c-di-GMP levels in surface-associated cells.

(**a**) Measurement of c-di-GMP levels in single cells inside a flow cell using the biosensor. Lines indicate the median RFI values per time point, and the shaded areas represent the 95% confidence intervals obtained from the bootstrap sampling distribution of the median RFI values. Time 0 h corresponds roughly to when cells initially encounter the surface of the flow cell.

(**b**) Plot of the probability of c-di-GMP increase, calculated by comparing the bootstrap sampling distributions of the median RFI values between every time point (I_t) and the first (I_1). C-di-GMP is determined to be increasing if $I_t/I_1 \geq 1.05$.

(**c**) Representative overlay images of filtered-fluorescence and phase-contrast channels depicting labeled MSHA pili in surface-associated *mshA*^{T70C} strains. Images representative of 3 independent analyses. Scale bar = 2 μm.

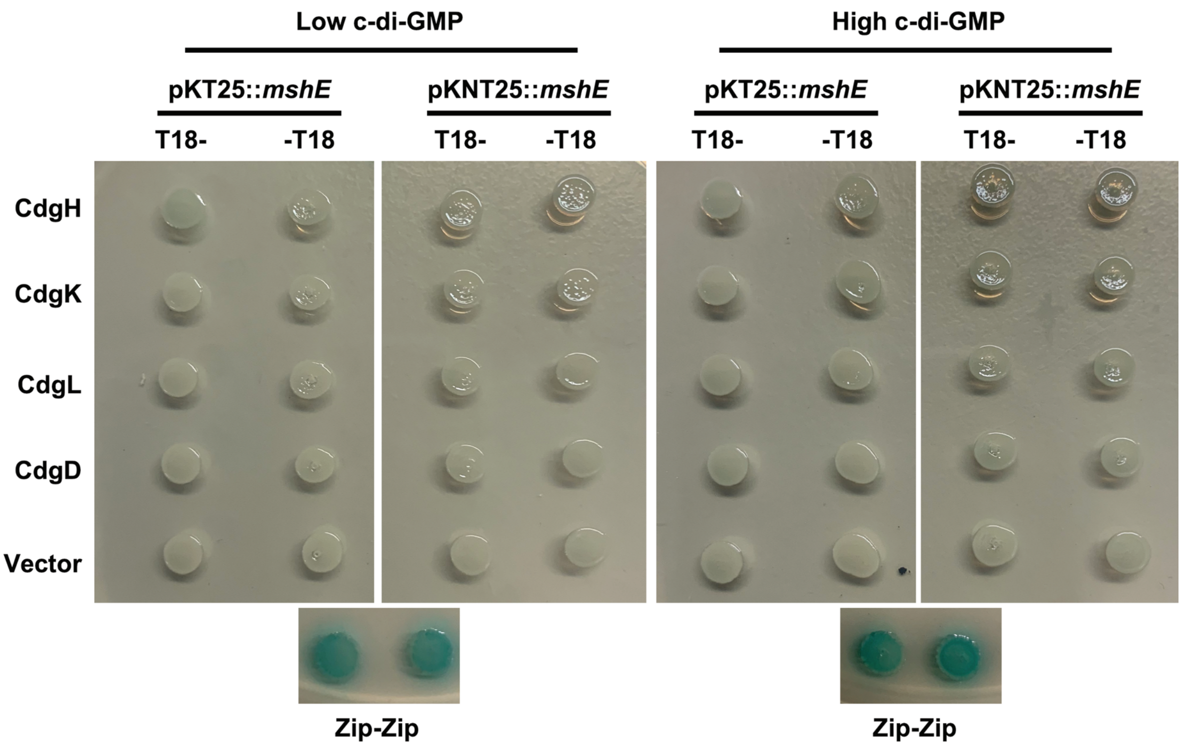

Supplementary Figure 8. Bacterial two-hybrid assays demonstrate that DGCs absent from the Δ4DGC strain do not interact directly with the MshE extension ATPase.
Representative images of bacterial two-hybrid assays examining for interactions of the DGCs CdgH, CdgK, CdgL, and CdgD with the MSHA extension ATPase MshE, under both low (BTH101) and high c-di-GMP levels (BTH101 *yhjH*::FRT). Images are representative from a total of three two-hybrid plasmid co-transformations.

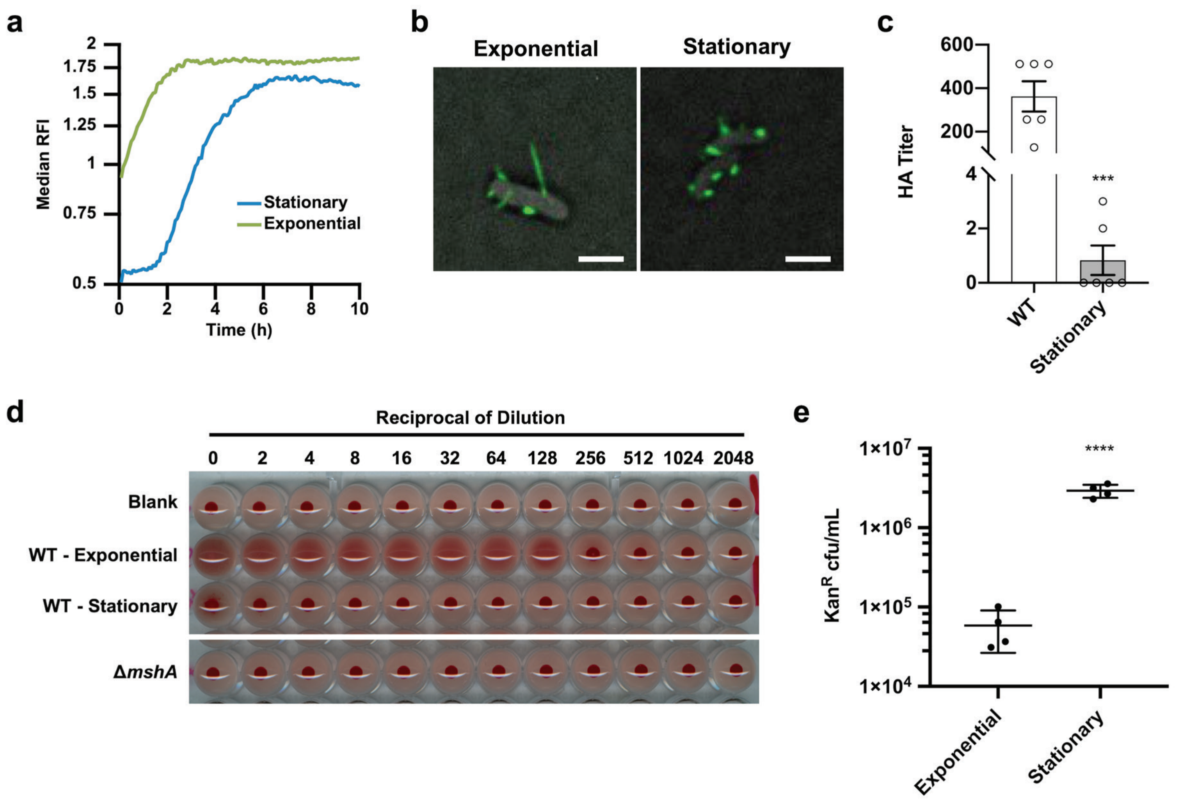

Supplementary Figure 9. MSHA levels are higher on the cell surface in exponential compared to stationary phase cells correlating with intracellular c-di-GMP levels.

(**a**) Measuring c-di-GMP levels of single cells inside a flow cell using the biosensor. Lines indicate the median RFI values per time point, and the shaded areas represent the 95% confidence intervals obtained from the bootstrap sampling distribution of the median RFI values. Time 0 h corresponds roughly to when cells initially encounter the surface of the flow cell.

(**b**) Representative overlay images of filtered-fluorescence and bright-field channels depicting labeled MSHA pili in surface-associated *mshA*^{T70C} strains. Images representative of 3 independent analyses. Scale bar = 2 μm.

(**c**) Analysis of surface MSHA production via hemagglutination (HA) assay. The reciprocal of the lowest fold dilution at which equivalent cell levels were able to agglutinate sheep erythrocytes (HA Titer) is plotted as the mean with error bars representing the SEM. For each strain n = 6 biological replicates were analyzed, with two technical replicates performed for each biological replicate. WT and Δ4DGC values are the same as shown in Figure 3e. ND = no observable hemagglutination at the highest cell concentration. Statistical analysis: each mutant HA titer was compared to WT via unpaired two-tailed Student's *t*-Test, ***$p = 0.0004$. Source data provided as a Source Data file.

(**d**) Representative output of HA assay presented in (c).

(**e**) VGJΦ phage transduction levels. Individual data points of kanamycin-resistant CFU/mL plotted with line at the mean and error bars representing the standard deviation. Biological replicates n = 4. Statistical analysis: unpaired two-tailed Student's *t*-Test, ****p = < 0.0001. Source data provided as a Source Data file.

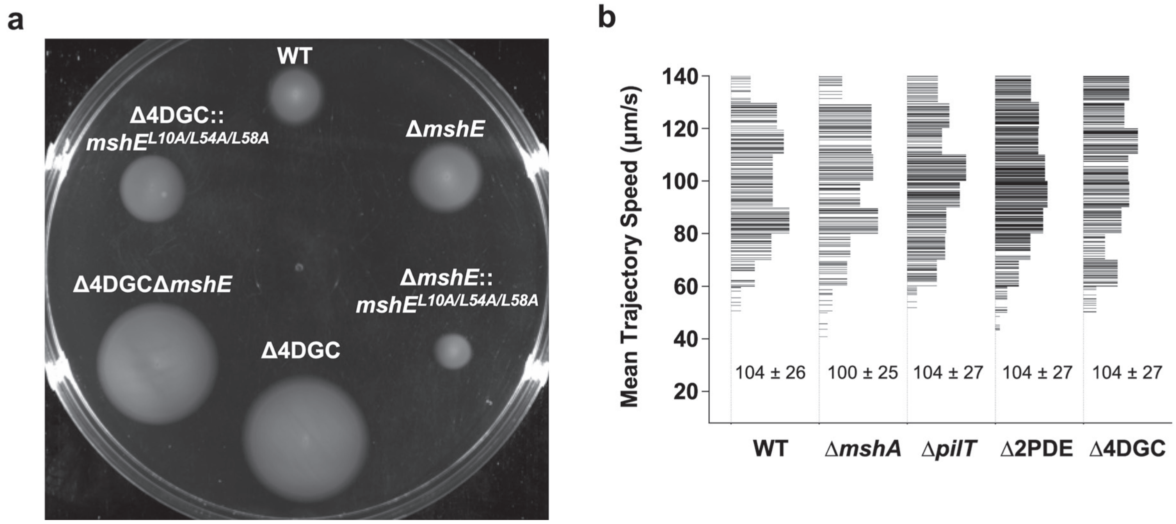

Supplementary Figure 10. Analysis of flagellar-based motility.

(**a**) Representative image of soft-agar swimming motility graphed in Figure 4c.

(**b**) High-speed microscopy analysis of bacterial swim speed in bulk media. Comparison between the mean trajectory speed (MTS) for WT, Δ*mshA*, Δ*pilT*, Δ2PDE, and Δ4DGC. Each horizontal line in the plot represents one trajectory of cells swimming freely in the bulk solution, imaged at ~100 mm above surface. The widths of the lines represent the distribution probabilities with a bin width of 10 μm per second. The mean of all trajectories ± the standard deviation for each strain is presented below the plot.

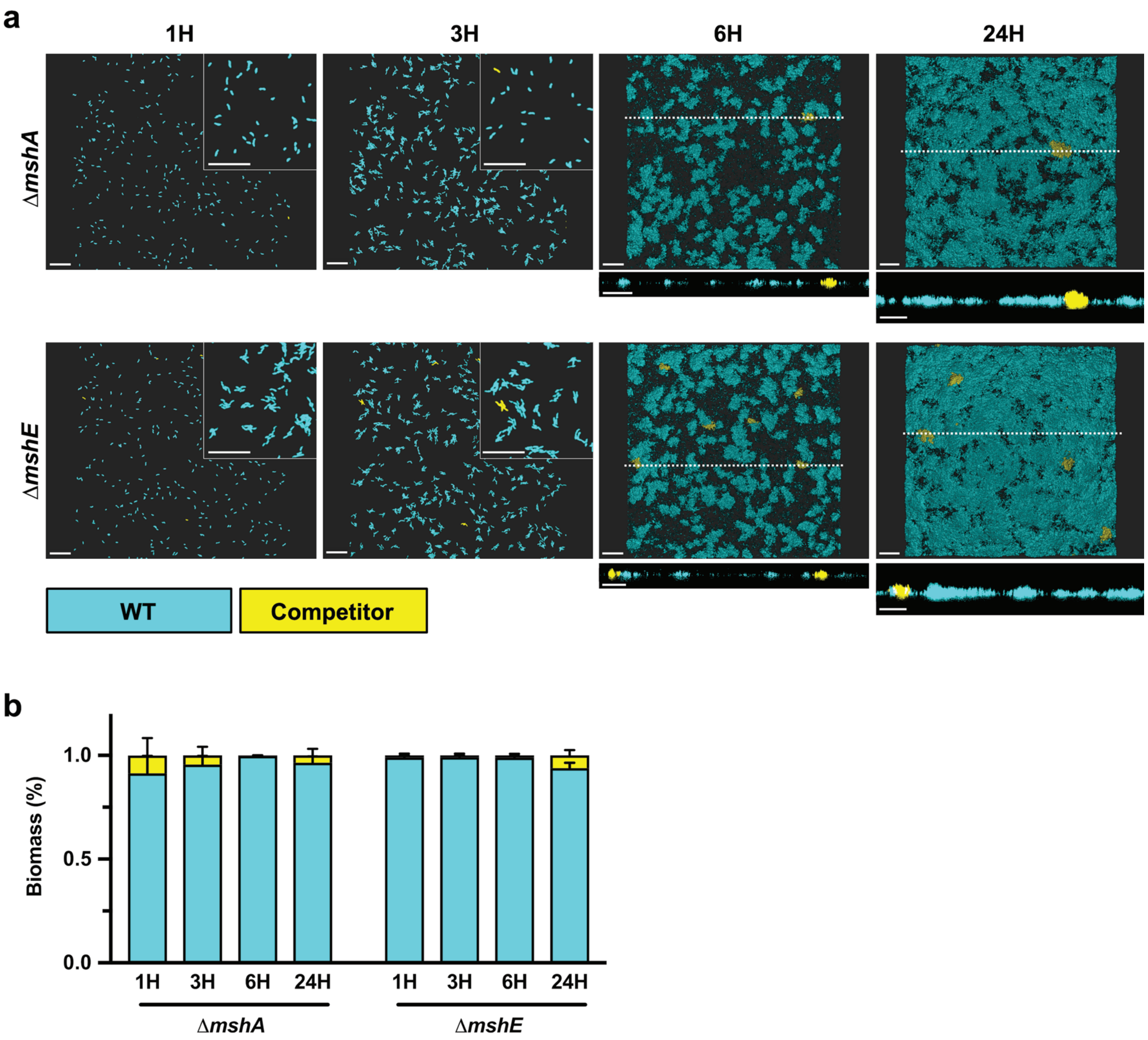

Supplementary Figure 11. MSHA extension and retraction dynamics dictate surface attachment and biofilm production in a model of competition.

a) Representative images of WT::RFP strain (cyan) vs. Δ*mshA*:: or Δ*mshE*::GFP strains in flow cell biofilm competition experiments at stages corresponding to the monolayer stage (1H), micro-colony formation (3H), three-dimensional micro-colony development (6-H), and mature biofilm (24H). Insets in the upper right corner of 1- and 3-hour images are zoomed in views obtained from the same image to depict single cells and micro-colonies. Images at 6- and 24-hours include a cross-sectional image of the XZ plane, and the position from which this cross-section was obtained is indicated in the image by the dashed line. Images representative of 2 independent analyses. Scale bars = 20 μm.

(**b**) WT::RFP (cyan) and competitor Δ*mshA*::Δ*mshE*::GFP (yellow) biomass levels presented as a percentage of the overall biomass. Biomass levels determined using Comstat2, and the percentage of the GFP-biomass from the whole was determined at each time point and plotted as the mean from two biological replicates imaged at 10x magnification (with three technical replicates per biological replicate and time point) with error bars indicating the standard error of the mean. All Comstat2 determined values are provided in Supplementary Table 2. Source data provided as a Source Data file.

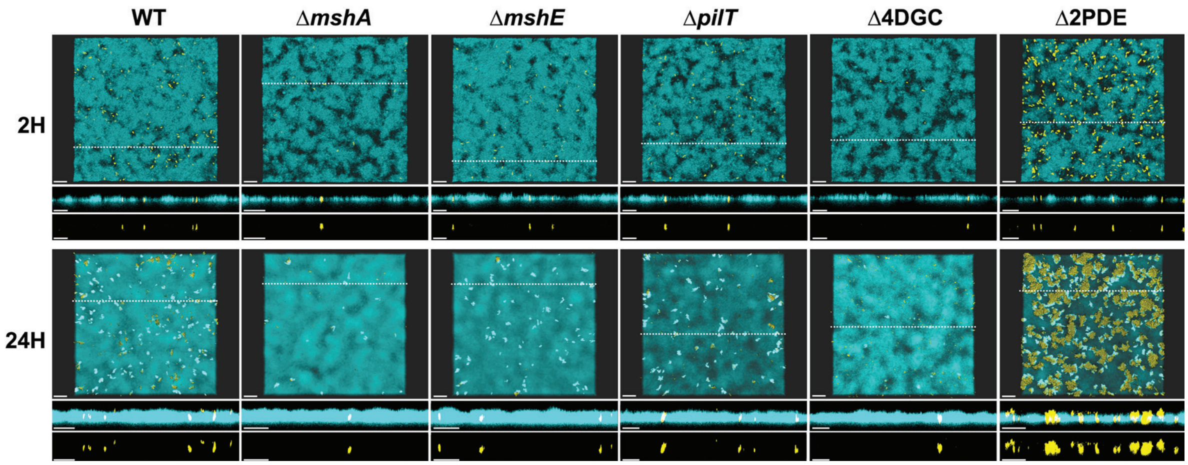

Supplementary Figure 12. MSHA production and proper dynamics correlates with fitness in a flow cell model of biofilm invasion.

Representative images of biofilm invasions, where invading strains (WT::/mutant::GFP, yellow) were injected into flow cell chambers containing a mature 24-hour WT::RFP biofilm (cyan), at 2- and 24-hours post-invasion. Images representative of 2 independent analyses. Images were generated using Imaris software. Images are representative from two biological replicates, with three technical replicate images obtained at 40x magnification per biological replicate. Scale bars = 20 μm. In order to see localization of the invading strain in relation to the mature WT::RFP biofilm, the opacity of the WT::RFP biofilm had to be increased in the full-size images. Cross-sections of the XZ planes (with both WT::RFP and mutant::GFP channels, or just the mutant::GFP channel) are shown below each image, and the dashed line in the above image indicates the position from which the cross-section image was obtained.

Strain / Treatment	Biomass ($\mu m^3/\mu m^2$)		Micro-colonies at Substratum			
			Total Number		Average Volume (μm^2)	
	Competitor	WT	Competitor	WT	Competitor	WT
**<u>1 Hour</u>**						
**WT vs. WT**	0.0383 (0.0009)	0.0345 (0.0037	0	0	n/a	n/a
**Δ*mshA* vs. WT**	0.0040 (0.0055)	0.0325 (0.0037)	0	0	n/a	n/a
**Δ*mshE* vs. WT**	0.0003 (0.0004)	0.0348 (0.0045)	0	0	n/a	n/a
**Δ*pilT* vs. WT**	0.0089 (0.0069)	0.0884 (0.0765)	0	0	n/a	n/a
**Δ4DGC vs. WT**	0.0633 (0.0107)	0.1229 (0.0087)	0	0	n/a	n/a
**Δ2PDE vs. WT**	0.0920 (0.0304)	0.1013 (0.0070)	0	0	n/a	n/a
**<u>3H Hour</u>**						
**WT vs. WT**	0.2659 (0.2556)	0.3479 (0.3772)	0	0	n/a	n/a
**Δ*mshA* vs. WT**	0.0180 (0.0251)	0.0787 (0.0005)	0	0	n/a	n/a
**Δ*mshE* vs. WT**	0.0007 (0.0009)	(0.0826 (0.0021)	0	0	n/a	n/a
**Δ*pilT* vs. WT**	0.0410 (0.0200)	0.4308 (0.0330)	0	0	n/a	n/a
**Δ4DGC vs. WT**	0.0927 (0.0399)	0.3530 (0.0024)	0	0	n/a	n/a
**Δ2PDE vs. WT**	0.3356 (0.1630)	0.3408 (0.0051)	2	0	343.29 (17.55)	n/a
**<u>6H Hour</u>**						
**WT vs. WT**	10.71 (1.82)	9.01 (0.28)	753	230	670.32 (101.93)	503.72 (219.79)
**Δ*mshA* vs. WT**	0.03 (0.01)	9.82 (3.93)	0	362	n/a	472.23 (46.25)
**Δ*mshE* vs. WT**	0.12 (0.12)	11.47 (0.60)	0	338	n/a	833.61 (409.67)
**Δ*pilT* vs. WT**	1.41 (0.87)	12.79 (3.37)	8	231	321.92 (34.12)	753.58 (334.20)
**Δ4DGC vs. WT**	2.07 (0.44)	15.73 (0.01)	10	195	378.07 (3.47)	792.26 (371.51)
**Δ2PDE vs. WT**	16.13 (3.34)	11.93 (0.62)	297	67	905.59 (5.78)	634.07 (133.06)
**<u>24H Hour</u>**						
**WT vs. WT**	35.76 (9.94)	20.79 (2.83)	-	-	-	-
**Δ*mshA* vs. WT**	1.06 (1.36)	23.83 (3.12)	-	-	-	-
**Δ*mshE* vs. WT**	1.67 (1.05)	25.12 (0.79)	-	-	-	-
**Δ*pilT* vs. WT**	4.93 (0.55)	22.57 (0.12)	-	-	-	-
**Δ4DGC vs. WT**	8.05 (10.99)	25.01 (2.86)	-	-	-	-
**Δ2PDE vs. WT**	41.36 (3.54)	11.70 (0.44)	-	-	-	-

Supplementary Table 1. Comstat2 quantitative values determined for flow cell biofilm competitions. Data presented as the mean (standard deviation) of two biological replicates, with three technical replicate images obtained at 10x magnification for each biological replicate

Strain/Plasmid	Relevant Genotype	Antibiotic^R	Source
	E. coli **Strains**		
S17-1 (λ*pir*)	*recA, thi, pro*, RP4-2-Tc::Mu-Km::Tn7 λ*pir* r_{K-} m_{K+} π_+	Tp_R, Sm_R	(1, 2)
SM10 (λ*pir*)	*Thi, thr, leu, tonA, lacY, supE, recA,* RP4-2-Tc::Mu λ*pir* π_+	Km_R	(1)
	V. cholerae **Strains**		
FY_Vc_00001	*Vibrio cholerae* El Tor Strain A1552	Rif_R	(3)
FY_Vc_12502	FY_Vc_00001 Δ*mshA*	Rif_R	(4)
FY_Vc_12677	FY_Vc_12502::*mshA*$_{T70C}$	Rif_R	This Study
FY_Vc_15283	FY_Vc_00001 Δ*pilA* Δ*tcpA* Δ*flaA* Δ*vpsII*	Rif_R	This Study
FY_Vc_15284	FY_Vc_15283 Δ*pilA* Δ*tcpA* Δ*flaA* Δ*vpsII* Δ*mshA*	Rif_R	This Study
FY_Vc_15285	FY_Vc_15283 Δ*pilA* Δ*tcpA* Δ*flaA* Δ*vpsII* Δ*mshE*	Rif_R	This Study
FY_Vc_15286	FY_Vc_15283 Δ*pilA* Δ*tcpA* Δ*flaA* Δ*vpsII* Δ*pilT*	Rif_R	This Study
FY_Vc_15287	FY_Vc_15283 Δ*pilA* Δ*tcpA* Δ*flaA* Δ*vpsII* Δ*pilU*	Rif_R	This Study
FY_Vc_15306	FY_Vc_15283 Δ*pilA* Δ*tcpA* Δ*flaA* Δ*vpsII* Δ*pilT* Δ*pilU*	Rif_R	This Study
FY_Vc_15290	FY_Vc_12677 *mshA*$_{T70C}$ Δ*mshE*	Rif_R	This Study
FY_Vc_15291	FY_Vc_15920 *mshA*$_{T70C}$ Δ*mshE*::*mshE*$_{G11I}$	Rif_R	This Study
FY_Vc_15292	FY_Vc_15920 *mshA*$_{T70C}$ Δ*mshE*::*mshE*$_{L10A/L54A/L58A}$	Rif_R	This Study
FY_Vc_15293	FY_Vc_12677 *mshA*$_{T70C}$Δ*pilT*	Rif_R	This Study
FY_Vc_15294	FY_Vc_12677 *mshA*$_{T70C}$Δ*pilU*	Rif_R	This Study
FY_Vc_15295	FY_Vc_12677 *mshA*$_{T70C}$Δ*pilT* Δ*pilU*	Rif_R	This Study
FY_Vc_8812	FY_Vc_00001 Δ*mshE*	Rif_R	(4)
FY_Vc_12451	FY_Vc_8812 Δ*mshE*::*mshE*$_{G11I}$	Rif_R	This Study
FY_Vc_12455	FY_Vc_8812 Δ*mshE*::*mshE*$_{L10A/L54A/L58A}$	Rif_R	This Study
FY_Vc_8802	FY_Vc_00001 Δ*pilT*	Rif_R	(4)
FY_Vc_8805	FY_Vc_00001 Δ*pilU*	Rif_R	(4)
FY_Vc_8807	FY_Vc_00001 Δ*pilT* Δ*pilU*	Rif_R	This Study
FY_Vc_15296	FY_Vc_12677 *mshA*$_{T70C}$ Δ*cdgH*	Rif_R	This Study
FY_Vc_15297	FY_Vc_12677 *mshA*$_{T70C}$ Δ*cdgK*	Rif_R	This Study
FY_Vc_15298	FY_Vc_12677 *mshA*$_{T70C}$ Δ*cdgL*	Rif_R	This Study
FY_Vc_15299	FY_Vc_12677 *mshA*$_{T70C}$ Δ*cdgD*	Rif_R	This Study
FY_Vc_15300	FY_Vc_6347 *mshA*$_{T70C}$ Δ*cdgH* Δ*cdgK* Δ*cdgL* Δ*cdgD* (Δ4DGC)	Rif_R	This Study
FY_Vc_1592	FY_Vc_00001 Δ*cdgH*	Rif_R	(5)
FY_Vc_1539	FY_Vc_00001 Δ*cdgK*	Rif_R	(5)
FY_Vc_162	FY_Vc_00001 Δ*cdgL*	Rif_R	(5)
FY_Vc_352	FY_Vc_00001 Δ*cdgD*	Rif_R	(6)
FY_Vc_6347	FY_Vc_00001 Δ*cdgH* Δ*cdgK* Δ*cdgL* Δ*cdgD* (Δ4DGC)	Rif_R	(7)
FY_Vc_15301	FY_Vc_12677 *mshA*$_{T70C}$Δ*cdgJ* Δ*rocS* (Δ2PDE)	Rif_R	This Study

FY_Vc_7150	FY_Vc_12677 Δ*cdgJ* Δ*rocS* (Δ2PDE)	Rif$_R$	This Study
FY_Vc_15302	FY_Vc_15300 *mshA*$_{T70C}$Δ4DGC Δ*mshE*	Rif$_R$	This Study
FY_Vc_15303	FY_Vc_15302 *mshA*$_{T70C}$Δ4DGC Δ*mshE*::*mshE*$_{L10A/L54A/L58A}$	Rif$_R$	This Study
FY_Vc_15304	FY_Vc_12677 *mshA*$_{T70C}$::mTn7-GFP	Rif$_R$	This Study
FY_Vc_5000	FY_Vc_00001 A1552::mTn7-RFP	Rif$_R$, Gm$_R$	This Study
FY_Vc_9573	FY_Vc_00001 A1552::mTn7-GFP	Rif$_R$, Gm$_R$	(8)
FY_Vc_9575	FY_Vc_00001 Δ*mshA*::mTn7-GFP	Rif$_R$, Gm$_R$	(4)
FY_Vc_9586	FY_Vc_00001 Δ*mshE*::mTn7-GFP	Rif$_R$, Gm$_R$	(4)
FY_Vc_9584	FY_Vc_00001 Δ*pilT*::mTn7-GFP	Rif$_R$, Gm$_R$	(4)
FY_Vc_13161	FY_Vc_00001 Δ4DGC::mTn7-GFP	Rif$_R$, Gm$_R$	This Study
FY_Vc_9591	FY_Vc_00001 Δ2PDE::mTn7-GFP	Rif$_R$, Gm$_R$	This Study
FY_Vc_1185	FY_Vc_00001 Δ*mshQ*	Rif$_R$	This Study
FY_Vc_15305	FY_Vc_12677 *mshA*$_{T70C}$Δ*mshQ*	Rif$_R$	This Study
SAD030	*Vibrio cholerae* El Tor Strain E7946	Sm$_R$	(9)
SAD033	SAD030 ΔVC1807	Sm$_R$, Kan$_R$	(9)
TND0473	SAD030 Δ*mshA*	Sm$_R$, Cm$_R$	(9)
CAH764	SAD030::*mshA*$_{T70C}$	Sm$_R$, Kan$_R$	(9)
HKQ001	CAH764 Δ*pilT*	Sm$_R$, Kan$_R$, Tm$_R$	(9)
TND1157	CAH764 Δ*pilU*	Sm$_R$, Kan$_R$, Tm$_R$	(9)
JLC419	CAH764 Δ*pilT*Δ*pilU*	Sm$_R$, Kan$_R$, Tm$_R$	(9)
HKQ006	CAH764 Δ*pilT*::*pilT*$_{K136A}$	Sm$_R$, Spec$_R$, Tm$_R$	(9)
TND1093	CAH764 Δ*pilU*::*pilU*$_{K134A}$	Sm$_R$, Spec$_R$, Tm$_R$	(9)
TND1095	CAH764 Δ*pilT*Δ*pilU*::*pilT*$_{K136A}$*pilU*$_{K134A}$	Sm$_R$, Spec$_R$, Tm$_R$	(9)
JLC446	CAH764 Δ*pilU*::*pilT*$_{K136A}$	Sm$_R$, Kan$_R$, Tm$_R$	(9)

Plasmids

pGP704-*sacB*28	pGP704 derivative, *mob/oriT sacB*	Amp$_R$	G. Schoolnik
pFY4874	pGP704-*sacB*28::Δ*mshA*	Amp$_R$	This Study
pFY4944	pGP704-*sacB*28::*mshA*$_{T70C}$	Amp$_R$	This Study
pFY1967	pGP704-*sacB*28::Δ*mshE*	Amp$_R$	(4)
pFY4839	pGP704-*sacB*28::*mshE*$_{G11I}$	Amp$_R$	This Study
pFY4843	pGP704-*sacB*28::*mshE*$_{L10A/L54A/L58A}$	Amp$_R$	This Study
pFY0399	pGP704-*sacB*28::Δ*cdgH*	Amp$_R$	(5)
pFY0405	pGP704-*sacB*28::Δ*cdgK*	Amp$_R$	(5)
pFY0241	pGP704-*sacB*28::Δ*cdgL*	Amp$_R$	(5)
pFY0157	pGP704-*sacB*28::Δ*cdgD*	Amp$_R$	(6)
pFY0386	pGP704-*sacB*28::Δ*cdgJ*	Amp$_R$	(5)
pFY0161	pGP704-*sacB*28::Δ*rocS*	Amp$_R$	(5)

pFY1976	pGP704-*sacB*28::Δ*pilT*	AmpR	(4)
pFY1979	pGP704-*sacB*28::Δ*pilU*	AmpR	(4)
pFY1982	pGP704-*sacB*28::Δ*pilT*Δ*pilU*	AmpR	(4)
pFY292	pGP704-*sacB*28::Δ*pilA*	AmpR	This Study
pFY0222	pGP704-*sacB*28::Δ*tcpA*	AmpR	This Study
pFY0332	pGP704-*sacB*28::Δ*flaA*	AmpR	(7)
pFY0659	pGP704-*sacB*28::Δ*vpsII*	AmpR	(10)
pFY4334	pGP704-*sacB*28::Δ*mshQ*	AmpR	This Study
pMCM11	pGP704::mTn7-GFP	GmR, AmpR	G. Schoolnik
pFY4535	pMMB67EH with Bc3-5 c-di-GMP biosensor and *hok/sok* region of pXB300	GmR	(11)

Supplementary Table 2. Bacterial strains and plasmids used in this study.

Supplementary References:

1. Simon, R., Priefer, U. & Pühler, A. A Broad Host Range Mobilization System for *In Vivo* Genetic Engineering: Transposon Mutagenesis in Gram Negative Bacteria. *Bio/Technology* **1**, 784–791 (1983).

2. de Lorenzo, V. & Timmis, K. N. B. T.-M. in E. [31] Analysis and construction of stable phenotypes in gram-negative bacteria with Tn5- and Tn10-derived minitransposons. in *Bacterial Pathogenesis Part A: Identification and Regulation of Virulence Factors* **235**, 386–405 (Academic Press, 1994).

3. Yildiz, F. H. & Schoolnik, G. K. *Vibrio cholerae* O1 El Tor: Identification of a gene cluster required for the rugose colony type, exopolysaccharide production, chlorine resistance, and biofilm formation. *Proc. Natl. Acad. Sci.* **96**, 4028 LP – 4033 (1999).

4. Jones, C. J. *et al.* C-di-GMP Regulates Motile to Sessile Transition by Modulating MshA Pili Biogenesis and Near-Surface Motility Behavior in *Vibrio cholerae*. *PLOS Pathog.* **11**, 1–27 (2015).

5. Shikuma, N. J., Fong, J. C. N. & Yildiz, F. H. Cellular levels and binding of c-di-GMP control subcellular localization and activity of the *Vibrio cholerae* transcriptional regulator VpsT. *PLoS Pathog.* **8**, (2012).

6. Lim, B., Beyhan, S., Meir, J. & Yildiz, F. H. Cyclic-diGMP signal transduction systems in *Vibrio cholerae*: Modulation of rugosity and biofilm formation. *Mol. Microbiol.* **60**, 331–348 (2006).

7. Liu, X., Beyhan, S., Lim, B., Linington, R. G. & Yildiz, F. H. Identification and Characterization of a Phosphodiesterase That Inversely Regulates Motility and Biofilm Formation in *Vibrio cholerae*. *J. Bacteriol.* **192**, 4541 LP – 4552 (2010).

8. Beyhan, S., Tischler, A. D., Camilli, A. & Yildiz, F. H. Differences in Gene Expression between the Classical and El Tor Biotypes of *Vibrio cholerae* O1. *Infect. Immun.* **74**, 3633 LP – 3642 (2006).

9. Chlebek, J. L. *et al.* PilT and PilU are homohexameric ATPases that coordinate to retract type IVa pili. *PLOS Genet.* **15**, e1008448 (2019).

10. Fong, J. C. N., Syed, K. A., Klose, K. E. & Yildiz, F. H. Role of *Vibrio* polysaccharide (vps) genes in VPS production, biofilm formation and *Vibrio cholerae* pathogenesis. *Microbiology* **156**, 2757–2769 (2010).

11. Zamorano-Sánchez, D. *et al.* Functional Specialization in *Vibrio cholerae* Diguanylate Cyclases: Distinct Modes of Motility Suppression and c-di-GMP Production. *MBio* **10**, e00670-19 (2019).
