# Supplementary material for: c-di-GMP modulates type IV MSHA pilus retraction and surface attachment in Vibrio cholerae
Source: Nat Commun. 2020 Mar 25;11:1549. doi: 10.1038/s41467-020-15331-8 (PMC7096442; doi:10.1038/s41467-020-15331-8)
Supplement: Supplementary file 2 — Description of Additional Supplementary Files [file 41467_2020_15331_MOESM2_ESM.pdf]

Description of Additional Supplementary Files

File Name: Supplementary Movie 1
Description: Three-dimensional reconstruction of cryo-ET data demonstrating MSHA distribution on the cell surface.

File Name: Supplementary Movie 2
Description: Video of time-lapse visualization of MSHA retraction shown in Figure 2a. Video shows overlay of digitally filtered fluorescence and bright-field microscopy images collected every 6 seconds over 10 minutes, played back at 5 frames per second (fps).

File Name: Supplementary Movie 3
Description: Video of MSHA retraction shown in Supplementary Figure 1d. Video shows overlay of digitally filtered fluorescence and bright-field microscopy images collected every 6 seconds over 10 minutes, played back at 5 frames per second (fps).

File Name: Supplementary Movie 4

Description: Video of MSHA retraction in the $mshE^{L10A/L54A/L58A}$ strain. Video shows overlay of digitally filtered fluorescence and bright-field microscopy images collected every 6 seconds over 10 minutes, played back at 10 frames per second (fps).

File Name: Supplementary Movie 5

Description: Video of time-lapse visualization of MSHA extension from Supplementary Figure 1e. Video shows overlay of digitally filtered fluorescence and bright-field microscopy images collected every 6 seconds over 6 minutes, played back at 5 frames per second (fps).
