# Supplementary material for: c-di-GMP modulates type IV MSHA pilus retraction and surface attachment in Vibrio cholerae
Source: Nat Commun. 2020 Mar 25;11:1549. doi: 10.1038/s41467-020-15331-8 (PMC7096442; doi:10.1038/s41467-020-15331-8)
Supplement: Supplementary file 8 — Reporting Summary [file 41467_2020_15331_MOESM8_ESM.pdf]

nature research

Corresponding author(s): Fitnat Yildiz and Gerard Wong

Last updated by author(s): 2/20/2020

Reporting Summary

Nature Research wishes to improve the reproducibility of the work that we publish. This form provides structure for consistency and transparency in reporting. For further information on Nature Research policies, see Authors & Referees and the Editorial Policy Checklist .

Statistics

For all statistical analyses, confirm that the following items are present in the figure legend, table legend, main text, or Methods section.

n/a	Confirmed	
☐	☒	The exact sample size (n) for each experimental group/condition, given as a discrete number and unit of measurement
☐	☒	A statement on whether measurements were taken from distinct samples or whether the same sample was measured repeatedly
☐	☒	The statistical test(s) used AND whether they are one- or two-sided<br>*Only common tests should be described solely by name; describe more complex techniques in the Methods section.*
☐	☒	A description of all covariates tested
☐	☒	A description of any assumptions or corrections, such as tests of normality and adjustment for multiple comparisons
☐	☒	A full description of the statistical parameters including central tendency (e.g. means) or other basic estimates (e.g. regression coefficient) AND variation (e.g. standard deviation) or associated estimates of uncertainty (e.g. confidence intervals)
☐	☒	For null hypothesis testing, the test statistic (e.g. F, t, r) with confidence intervals, effect sizes, degrees of freedom and P value noted<br>*Give P values as exact values whenever suitable.*
☒	☐	For Bayesian analysis, information on the choice of priors and Markov chain Monte Carlo settings
☒	☐	For hierarchical and complex designs, identification of the appropriate level for tests and full reporting of outcomes
☒	☐	Estimates of effect sizes (e.g. Cohen's d, Pearson's r), indicating how they were calculated

Our web collection on statistics for biologists contains articles on many of the points above.

Software and code

Policy information about availability of computer code

Data collection	No custom data collection software not previously described was used for these studies.
Data analysis	Data analysis performed with the following softwares, all of which are publicly available:<br>Image J Fiji Version 2.0.0-rc69/1.52p<br>TOMO3D<br>IMOD<br>UCSF Chimera<br>GraphPad Prism Version 8.3.1<br>BioRad Image Lab Version 5.2.1 build 11<br>MATLAB R2019a<br>Comstat2<br>Imaris Version 9.3.0

For manuscripts utilizing custom algorithms or software that are central to the research but not yet described in published literature, software must be made available to editors/reviewers. We strongly encourage code deposition in a community repository (e.g. GitHub). See the Nature Research guidelines for submitting code & software for further information.

Data

Policy information about availability of data

All manuscripts must include a data availability statement. This statement should provide the following information, where applicable:

- Accession codes, unique identifiers, or web links for publicly available datasets
- A list of figures that have associated raw data
- A description of any restrictions on data availability

The source data underlying Figures 2b-d, 2f, 3a-b, 3e, 3g, 4b-e, 7b, Supplementary Figures 1b, 4, 5b-c, 9c, 9e, and 11b are provided as a Source Data file. The source data for all other figures is available upon request from the corresponding authors.

Field-specific reporting

Please select the one below that is the best fit for your research. If you are not sure, read the appropriate sections before making your selection.

- [x] Life sciences
- [ ] Behavioural & social sciences
- [ ] Ecological, evolutionary & environmental sciences

For a reference copy of the document with all sections, see nature.com/documents/nr-reporting-summary-flat.pdf

Life sciences study design

All studies must disclose on these points even when the disclosure is negative.

Sample size	No statistical method was used to determine sample sizes, as sample sizes were chose based upon historical data.<br><br>Single-cell experiment sample sizes were determined as previously published in the following references:<br>Lee, C. K. et al. Multigenerational memory and adaptive adhesion in early bacterial biofilm communities. Proc. Natl. Acad. Sci. 115, 4471–4476 (2018).<br>Zamorano-Sánchez, D. et al. Functional Specialization in Vibrio cholerae Diguanylate Cyclases: Distinct Modes of Motility Suppression and c-di-GMP Production. MBio 10, e00670-19 (2019).
Data exclusions	No data were excluded from these analyses.
Replication	All analyses were replicated with at least two biological replicates, or more where indicated, and replication attempts were successful.
Randomization	There was no randomization for these experiments. All strains were grown under similar conditions, to ensure that bacteria were at equivalent states at the beginning of each experiment. Therefore, all observed differences are attributed to either the genotype differences between the strains being tested, or the variations in the treatment conditions tested on the strains.
Blinding	Blinding was not used, for as mentioned above, all strains were grown under similar conditions prior to the start of experiments, and the observed differences are attributed to either the genotype or treatment of the strain under the experimental conditions tested.

Reporting for specific materials, systems and methods

We require information from authors about some types of materials, experimental systems and methods used in many studies. Here, indicate whether each material, system or method listed is relevant to your study. If you are not sure if a list item applies to your research, read the appropriate section before selecting a response.

Materials & experimental systems

n/a	Involved in the study	
[ ]	[x]	Antibodies
[x]	[ ]	Eukaryotic cell lines
[x]	[ ]	Palaeontology
[x]	[ ]	Animals and other organisms
[x]	[ ]	Human research participants
[x]	[ ]	Clinical data

Methods

n/a	Involved in the study	
[x]	[ ]	ChIP-seq
[x]	[ ]	Flow cytometry
[x]	[ ]	MRI-based neuroimaging

Antibodies

Antibodies used	Rabbit polyclonal anti-MshA was used at a 1:1000 dilution of 1mg/mL stock for immunoblot experiments and 40ug total for Cryo-ET analysis.<br>HRP-conjugated goat anti-rabbit antibody was used for immunoblots at 1:2500 dilution of 2mg/mL stock (Promega Co.)

Validation

Anti-MshA antibody does not recognize any bands in a strain lacking the gene encoding for MshA.
